# Gelatin coating enhances therapeutic cell adhesion to the infarcted myocardium via ECM binding

**DOI:** 10.1371/journal.pone.0277561

**Published:** 2022-11-10

**Authors:** Kara A. Davis, Anuhya Gottipatti, Hsuan Peng, Renee Donahue, Lakshman Chelvarajan, Calvin Cahall, Himi Tripathi, Ahmed Al-Darraji, Shaojing Ye, Ahmed Abdel-Latif, Brad J. Berron

**Affiliations:** 1 Department of Chemical and Materials Engineering, University of Kentucky, Lexington, KY, United States of America; 2 Gill Heart and Vascular Institute and Division of Cardiovascular Medicine, University of Kentucky and the Lexington VA Medical Center, Lexington, KY, United States of America; 3 Division of Cardiovascular Medicine, Department of Internal Medicine, University of Michigan, Ann Arbor, Michigan, United States of America; Università degli Studi della Campania, ITALY

## Abstract

Acute myocardial infarction (AMI) results in weakening of the heart muscle and an increased risk for chronic heart failure. Therapeutic stem cells have been shown to reduce inflammatory signaling and scar tissue expansion, despite most of these studies being limited by poor retention of cells. Gelatin methacrylate (GelMA) coatings have been shown to increase the retention of these therapeutic cells near the infarct. In this work, we evaluate two different potential binding partners for GelMA-coated bone marrow cells (BMCs) and myocardial tissue: the extracellular matrix (ECM) and interstitial non-cardiomyocytes. While cells containing β1 integrins mediate cell-ECM adhesion *in vivo*, these cells do not promote binding to our collagen-degraded, GelMA coating. Specifically, microscopic imagining shows that even with high integrin expression, GelMA-coated BMCs do not bind to cells within the myocardium. Alternatively, BMC incubation with decellularized heart tissue results in higher adhesion of coated cells versus uncoated cells supporting our GelMA-ECM binding mode. To further evaluate the ECM binding mode, cells were incubated on slides modified with one of three different major heart ECM components: collagen, laminin, or fibronectin. While all three components promoted higher adhesion than unmodified glass, collagen-coated slides resulted in a significantly higher adhesion of GelMA-coated BMCs over laminin and fibronectin. Incubation with unmodified BMCs confirmed that without a GelMA coating minimal adhesion of BMCs occurred. We conclude that GelMA cellular coatings significantly increase the binding of cells to collagen within the ECM. Our results provide progress towards a biocompatible and easily translatable method to enhance the retention of transplanted cells in human studies.

## Introduction

Cardiovascular disease, including acute myocardial infarction (AMI), remains one of the leading causes of death worldwide [1, 2]. During an AMI, the myocardium undergoes irreversible damage to healthy tissue which is replaced by a fibrotic non-functional scar, thus leaving patients at increased risk for developing heart failure [3]. The majority of the damage caused to the heart tissue is caused by the body’s own immune response. For this reason, many have explored the use of bone marrow-derived stem cells (BMCs) that can regulate the body’s immune system to facilitate myocardial repair [4, 5]. However, many of these efforts have been limited due to cell retention of less than 1% [6].

Cell surface engineering has emerged as a novel approach for addressing cell adhesion issues in a wide variety of applications [7]. Specifically, our lab developed gelatin-based cellular coatings that allow for a 3-fold increase of therapeutic mesenchymal stem cells (MSCs) retention within the infarct myocardium [8]. Additionally, this increased retention of MSCs resulted in a significant reduction in scar size compared to mice treated with uncoated MSCs. Overall, the use of gelatin-based cellular coatings promotes long-term patient health and decreased risk of repeated cardiovascular disease.

**Fig 1:**
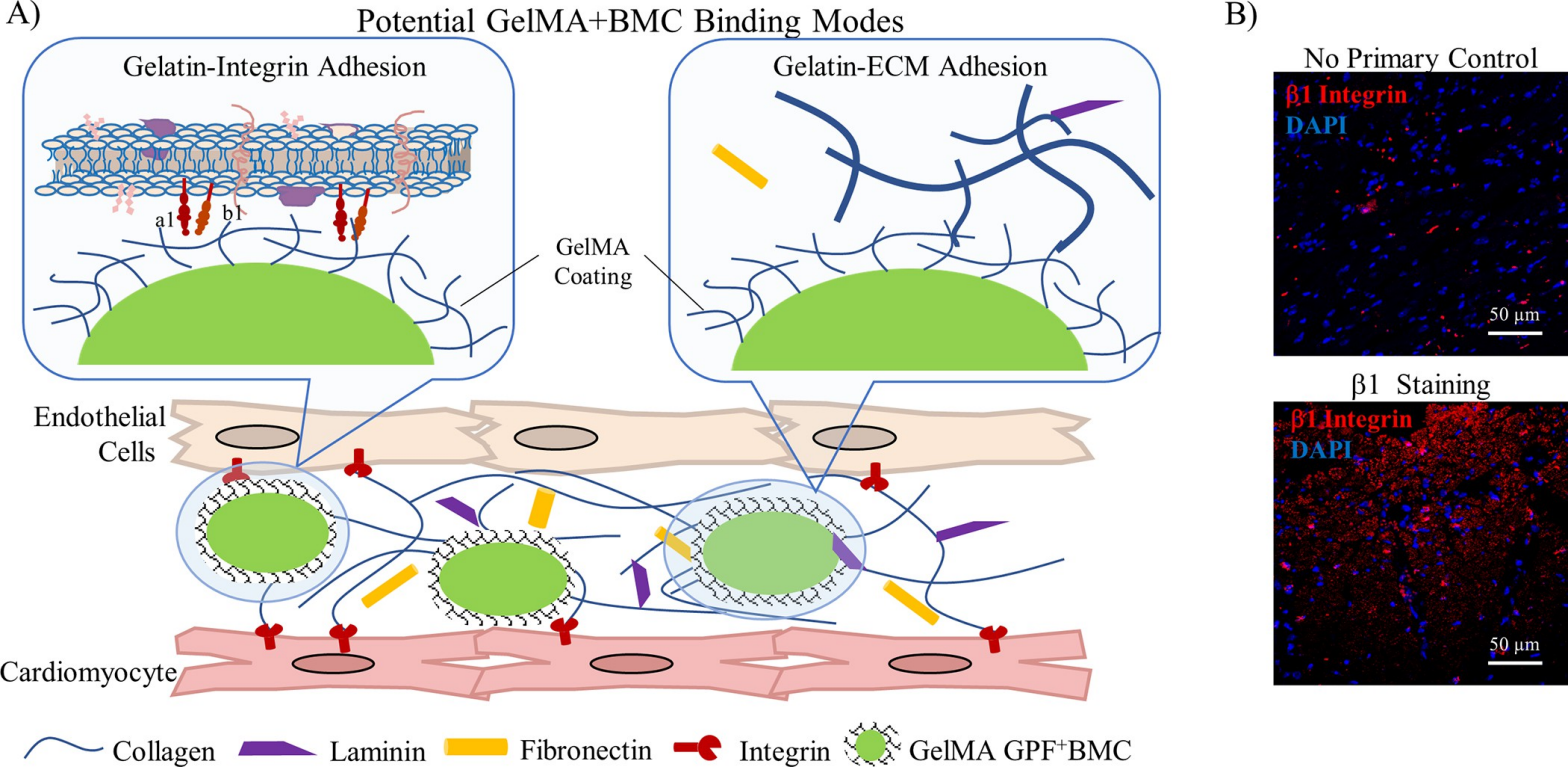
Integrins and ECM components serve as potential binding modes for GelMA coated BMC *in vivo*. A) Schematic of potential binding modes for GelMA coated BMC within the myocardium. B) Immunofluorescent imagining of infarct heart tissue stained with primary β1 integrin antibody followed by Donkey anti-rabbit Alexa Fluor 568. No primary antibody was used as a negative control. β1 integrin expression is shown in red with DAPI nuclear staining in blue (scale bar = 200 μm).

The gelatin-based coatings used in previous studies are made up of a PEG cross-linker and gelatin methacrylate (GelMA). One of the main advantages of using GelMA is its well-established production and regulatory landscape [9]. While our lab has already demonstrated an increased *in vivo* retention for coated cells over uncoated cells [8], the mechanism for GelMA adhesion *in vivo* has yet to be determined. The stem cell injection site consists of two major components: the extracellular matrix (ECM) and resident cells. Here, we compare two potential binding modes, gelatin-ECM and gelatin-cell, to determine how GelMA coating increases the retention of BMCs in the infarcted myocardium (Fig 1). Resident heart cells serve as potential binding sites for GelMA coating due to the interaction between β1 integrins and collagen [10]. The gelatin in the coatings is a degraded collagen and will likely retain many binding motifs for β1 integrins. To evaluate the role of gelatin-cell interactions, we evaluate the adhesion of GelMA-coated BMCs to two different cell lines of either high β1 expression or low β1 expression.

Alternatively, the possibility exists that gelatin in the coatings interacts with the ECM components of the myocardium. We first broadly probe gelatin-ECM adhesion by evaluating coated cell retention on decellularized (decell) heart tissue. We further investigate the role of specific ECM components in cell adhesion. Collagen is a good candidate for GelMA binding due to its prevalence in cardiac ECM [11]. We also evaluated laminin as a target ECM protein, and fibronectin due to the abundance of collagen-binding sites [11].

## Materials and methods

### Study design

Male C57BL/6J WT and C57BL/6-Tg(CAG-EGFP)1Osb/J mice (Jackson Laboratory, BarHarbor, ME, strain code 003291), aged 8–10 weeks, were used in this study. Mice were fed ad libitum with a normal chow diet (Harlan Teklad, 2018) and randomly assigned to experimental groups. All procedures were conducted under the approval of the University of Kentucky IACUC in accordance with the NIH Guide for the Care and Use of Laboratory Animals (DHHS publication No. [NIH] 85–23, rev. 1996). H9C2 (rat cardiac myoblast) were obtained from ATCC (CRL-1446). HUVEC (human umbilical vein endothelial cells) were obtained from Lonza (C2517A).

### Murine model of myocardial infarction and injection of BMC

Mice were anesthetized with 1–3% isoflurane using a small animal vaporizer system. We then examined pain prior to surgery to make sure that the mouse was adequately anesthetized. Using a small incision over the fourth intercostal space, the heart was exposed, ‘popped’ out of the thorax and the left anterior descending coronary artery (LAD) was ligated approximately 3 mm below the level of the left atrial appendage using a 6–0 silk suture as previously described [12, 13]. Immediately following LAD ligation, 3 x 10^6^ cells were injected in the infarct border at the anterior and posterior wall using 2 different injections (25 μL total) using a 25-gauge needle. After injection, the heart was returned to intrathoracic space and the air was manually evacuated before suturing the incision site. Buprenorphine SR (1.5 mg/kg, ZOOPharm) was administered once immediately after surgery. Animals were monitored for signs of pain, discomfort, or reduced food or water intake every 8 hours for 24 hours followed by daily monitoring for 1 week. If animals displayed any of these signs, additional doses of Buprenorphine SR were administered. Animals were also monitored for any signs of clinical deterioration or overt heart failure, and any animals that showed signs of decompensation were resuscitated, and if this was unsuccessful, they were humanly euthanized. We also monitored mice for additional signs of distress or weight loss >15–20% from baseline weight; hunched posture; ruffled coat; etc. If the mice exhibited any of these signs, they were euthanized without any subsequent experimental procedures. Most of the mortality in our myocardial infarction experimental group occurred in the first week after surgery. For the vast majority of mortality cases, necropsy showed blood filled thoracic cavity suggesting myocardial rupture as the cause of death.

The appearance of the surgical site was checked for signs of infection, loose sutures, and seroma formation. When possible, infection, sutures, or seroma formation is treated medically (antibiotics, if indicated) or surgically (replacing sutures or percutaneous drainage, respectively). In rare cases of infections, we consulted with the Division of Laboratory Animal Resources veterinary for advice, treatment, and additional advanced management. In cases where complications were felt to be causing the animal significant distress as detailed above, the animal was euthanized. Mice were euthanized using high-dose isoflurane followed by cervical dislocation to confirm their euthanasia. Surgery and cell injections were performed by a blinded surgeon.

### Generation of bone marrow derived cells

Total bone marrow cells were isolated from C57BL/6-Tg(CAG-EGFP)1Osb/J. Marrow from the femur, tibia and hip bones were flushed with cold phosphate-buffered saline (PBS) supplemented with 10% fetal bovine serum (FBS, VWR) using a 22-gauge needle. The collected bone marrow cell (BMC) suspension was then filtered through a 40μm strainer and pelleted with centrifugation at 500xg for 5mins. BMCs were then resuspended in 1x RBC lysis buffer (eBioscience™, cat# 00-4300-54) and incubated for 10 minutes at room temperature to remove red blood cells. The GFP^+^ BMCs were then washed as described above and resuspended in cold PBS and proceeded to cell coating immediately.

### BMC encapsulation

GFP^+^ BMCs were nonspecifically, covalently modified using a biotin-succinimidyl ester conjugate (NHS-biotin; EZ-link Sulfo-NHS-LC-Biotin, Thermo Fisher). NHS-biotin was used at a concentration of 1 mM in PBS for 40 min on ice with a volume of 500 μL per million cells. Following biotin conjugation, BMC were rinsed with PBS twice and aliquoted into 1.5 × 10^6^ cell samples. The cell pellet was then resuspended in 1 mL of PBS containing 35 μg/mL streptavidin-eosin isothiocyanate (SAEITC) and incubated for 30 min on ice. SAEITC conjugation was performed in-house, through formation of a thiourea bond [14]. Briefly, SA (SigmaAldrich), dissolved in a carbonate buffer, and eosin isothiocyanate (SigmaAldrich), dissolved in DMSO, were reacted at a 10:1 ratio for 8 hours at 4°C. The solution was then concentrated to 1 mg/mL in PBS and purified through Zeba spin desalting column.

The polymer mixture buffer was prepared using 1% PEGDA 3400, 35 mM triethanol amine, and 35 mM 1-vinyl-2-pyrrolidinone in PBS. PEGDA 3400 is added as a low Molecular Weight crosslinking agent to promote cell coating uniformity [8]. Next, 3 wt% gelatin methacrylate (Allevi) was added to the buffer and sonicated for 30 min. The final macromer solution (GelMA) was filtered through a 0.2 μm syringe filter and stored at 37°C. Following filtration, 350 μL of the polymer mixture was added to the cell pellet, gently vortexed and loaded into a chip-clip well (Whatman) with a standard microscopy slide. The chip-clip was incubated in a dark, N_2_ purged, sealed, zip lock bag for 3 min before polymerization. The cells were polymerized for 10 min at 30 mW/cm^2^ under 530 nm green light while purging with N_2_ followed by rinsing twice with 1 mL of PBS. To determine the presence of a GelMA coating, BMC were stained with primary anti-collagen 1 antibody (Abcam) followed by secondary Alexa Fluor 647 antibody (Invitrogen). Samples of 10,000 events were analyzed by flow cytometry, and the mean fluorescence for secondary antibody tagged cells was recorded. GFP^-^BMC were used for flow cytometry analysis to reduce bleed over of FITC channel.

### Flow cytometry analysis of heart tissue

Heart tissue was harvested at 7 days and placed in ice cold PBS instantly. Heart tissue was minced then digested using a Collagenase B (Roche) and Dispase II (Roche) solution for 30 minutes at 37°C with mixing every 5 minutes. The enzymatic reaction was stopped by dilution with Flow Buffer (PBS + 5% normal goat serum + 0.1% sodium azide) and the heart cell suspensions were passed through 40 μm cell strainers. Cells were centrifuged at 400×g for 5 min at 4°C, then suspended in flow buffer. Cells were incubated directly for 30 minutes with eFluor 660 conjugated GFP Antibody Clone 5F12.4 (eBioscience, Thermo Fisher Scientific) and APC-CY7-conjugated CD45 (Biolegend). After incubation, cells were washed twice using flow buffer and analyzed using an LSR II (Becton Dickinson) in the University of Kentucky Flow Cytometry Core. Laser calibration and compensation were carried out utilizing unstained and single fluorescent control beads (eBioscience). FlowJo v7 software was used to generate dot plots and analyze the data.

### Immunofluorescence analysis

Immunofluorescent assessments were carried out on de-paraffinized and rehydrated sections as previously described [13]. Briefly, sections were exposed to heat-mediated epitope retrieval in citrate buffer, pH 6.0 (Vector Laboratories, Burlingame CA)) for 20 mins, then blocked with normal goat serum for 10 minutes at 37°C. Slides were incubated with primary antibodies: rabbit anti-GFP (Abcam, Cambridge, United Kingdom) or rat anti-mouse CD68 (Abcam). After washing, sections were incubated with secondary antibodies conjugated to APC or FITC, respectively. The sections were finally incubated with Sudan Black B (Sigma Aldrich, St. Louis, MO) for 30 minutes. Adjacent areas in the peri-infarct and remote zones were analyzed (1 section/animal, n = 2 animals/group) at 40x magnification using Nikon Confocal Microscope A1 (Nikon, Tokyo, Japan) in the University of Kentucky Confocal Microscopy facility. Calculations were performed using the Cell Counter plugin for ImageJ, version 1.51d (NIH, Rockville, MD).

For Beta 1 integrin evaluation in infarcted tissue slices, immunohistochemical assessments were carried out on de-paraffinized and rehydrated sections as previously described [13]. Briefly, sections were exposed to heat-mediated epitope retrieval in tris EDTA buffer, pH 9.0 (Vector Laboratories, Burlingame CA)) for 20 mins, then blocked (3% normal donkey serum + 0.3% Triton X-100 in 1x PBS) for 30 minutes at room temperature. Slides were incubated with primary antibody (1.5% normal donkey serum +0.3% Triton X-100 in 1x PBS): rabbit anti-Beta 1 Integrin (Bioss, 1:500, #BS-0486R) overnight at 4˚C. After washing, sections were incubated with donkey anti-rabbit Alexa Fluor 568 (Abcam, 1:500, #ab175692) secondary antibody for 30 minutes at room temperature. Slides were washed and coverslipped with Vectastain mounting medium with DAPI (Vector # H-1200). Slides were imaged at 40X magnification on a Nikon AR1 confocal imaging system in the University of Kentucky Confocal Microscopy Core.

### Statistical analysis of *in vivo* and *in vitro* results

Values are expressed as mean ± standard error of mean (SEM). We used unpaired Student t-test or analysis of variance (one-way or multiple comparisons) to estimate differences, as appropriate. We utilized two-sided Dunnett or Dunn tests for post hoc multiple comparison procedures, with control samples as the control category. Throughout the analyses, a p-value less than 0.05 was considered statistically significant. All statistical analyses for *in vivo* studies were performed using the Prism 7 software package (GraphPad, La Jolla, CA).

### Cell β1 integrin staining and BMC adhesion

H9C2 cells were cultured in Dulbecco modified eagle medium (DMEM, VWR) supplemented with 10% fetal bovine serum (FBS, VWR) and 1% penicillin/streptomycin (VWR) at 37˚C and 5% CO_2_. HUVEC cells were cultured in EGM-2 BulletKit (Lonza) supplemented with 1% penicillin/streptomycin at 37˚C and 5% CO_2_. Cells were seeded in T-182 cm^2^ tissue culture flasks (VWR) and grown to approximately 80% confluence. Cells were aspirated using 0.25% Trypsin-EDTA 1X (VWR) and then resuspended in 5 mL medium to neutralize the trypsin. Cells were pelleted at 400xg at 4 ˚C for 3 min, washed three times in 1 mL PBS and then resuspended in PBS for experiments.

H9C2 and HUVEC cells were stained for the presence of β1 integrin using anti-β1 integrin (Bioss Antibodies) followed by secondary Alexa Fluor 647 antibody (Invitrogen). Samples of 10,000 events were analyzed by flow cytometry, and the mean fluorescence for secondary antibody-tagged cells was recorded. FlowJo v7 software was used to generate dot plots and analyze the data.

H9C2 and HUVEC cells were stained using cell tracker red (Invitrogen) and then cultured in 4-well culture slides (Thermo Fisher) at 37°C and 5% CO_2_ until they reached 90% confluency. Following incubation, media was removed, and cells were rinsed with PBS. Coated or uncoated GFP^+^ BMC were added to culture slides at 2.5 × 10^5^ cells/mL and allowed to incubate for 40 minutes. Following incubation, BMC adherence was evaluated by placing slides in a 50 mL centrifuge tube filled to the top with PBS and centrifuging for 5 minutes at 800xg. Images before and after centrifugation were captured with a Nikon Ti-U inverted microscope and used to quantify the retention of cells.

### Preparation of decellularized (Decell) tissue

A mouse heart was obtained from the University of Kentucky, department of animal and food sciences. The heart was frozen at -20°C for a minimum of 24 h prior to decellularization. After thawing, the heart was sectioned into pieces approximately 2 cm in diameter and 1 mm in thickness. These pieces were decellularized by room temperature immersion in 1% sodium dodecyl sulfate in deionized water mixed with 0.5% penicillin-streptomycin for 96 h, followed by 1% Triton X-100 in deionized water for 48 h, with solution replaced every 24 h. Tissue was continuously shaking on a shaker table. The scaffolds were washed with deionized water for two days. Decellularized heart tissue was confirmed by Hematoxylin and eosin staining and DNA quantification by PureLinkTM Genomic DNA Mini Kit (Thermo Fisher) according to the manufacturer’s protocol.

### Cell incubation with modified substrates

GFP^+^ BMCs were used in these studies to detect BMC within the ridges of decell tissue. GelMA-coated BMCs were allowed to incubate with glass substrates coated with either collagen (VWR), fibronectin (VWR), laminin (Gibco) or Decell Tissue for 40 minutes. Laminin-coated slides were prepared in-house by covering sterile glass microscope slides with 5 μg/mL laminin in PBS for 2 hours at 37°C. Following incubation, BMC adherence was evaluated by placing slides in a 50 mL centrifuge tube filled to the top with PBS and centrifuging for 5 minutes at 800xg. Images before and after centrifugation were captured with a Nikon Ti-U inverted microscope and used to quantify the retention of cells. BMC were imaged at 40x and counted using Image J to determine before and after cell counts. GFP+BMC were also imaged at 4x to verify the uniformity of adhesion across the substrate.

## Results

**Fig 2:**
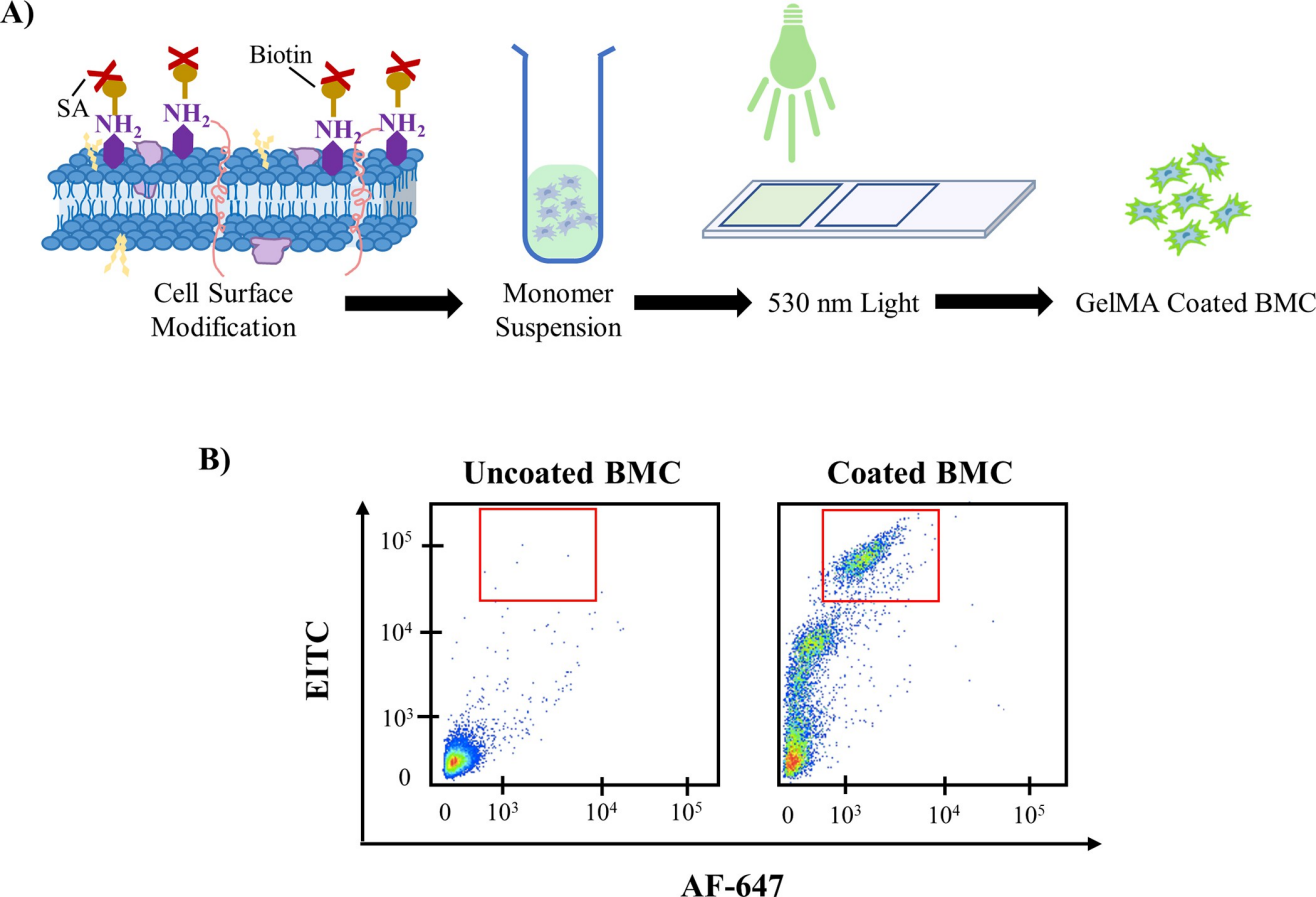
Flow cytometry coating analysis of BMC. A) Schematic of GelMA coating process through modification of cell surface amines. B) Flow cytometric analysis of coated versus uncoated BMCs stained with primary collagen antibody followed by secondary AF-647.

The coating strategy and analysis of the coated cells are shown in Fig 2. Modification of the GFP^+^ BMC’s peripheral membrane results from nonspecific biotinylation with sulfo-NHS-biotin, streptavidin-eosin incubation, and photopolymerization in a GelMA monomer solution under 530 nm light. Flow cytometric analysis shows a distinct eosin-positive and gelatin-positive population which accounts for 39% of cells (Fig 2B).

### Coated BMCs retention in the heart in vivo

**Fig 3:**
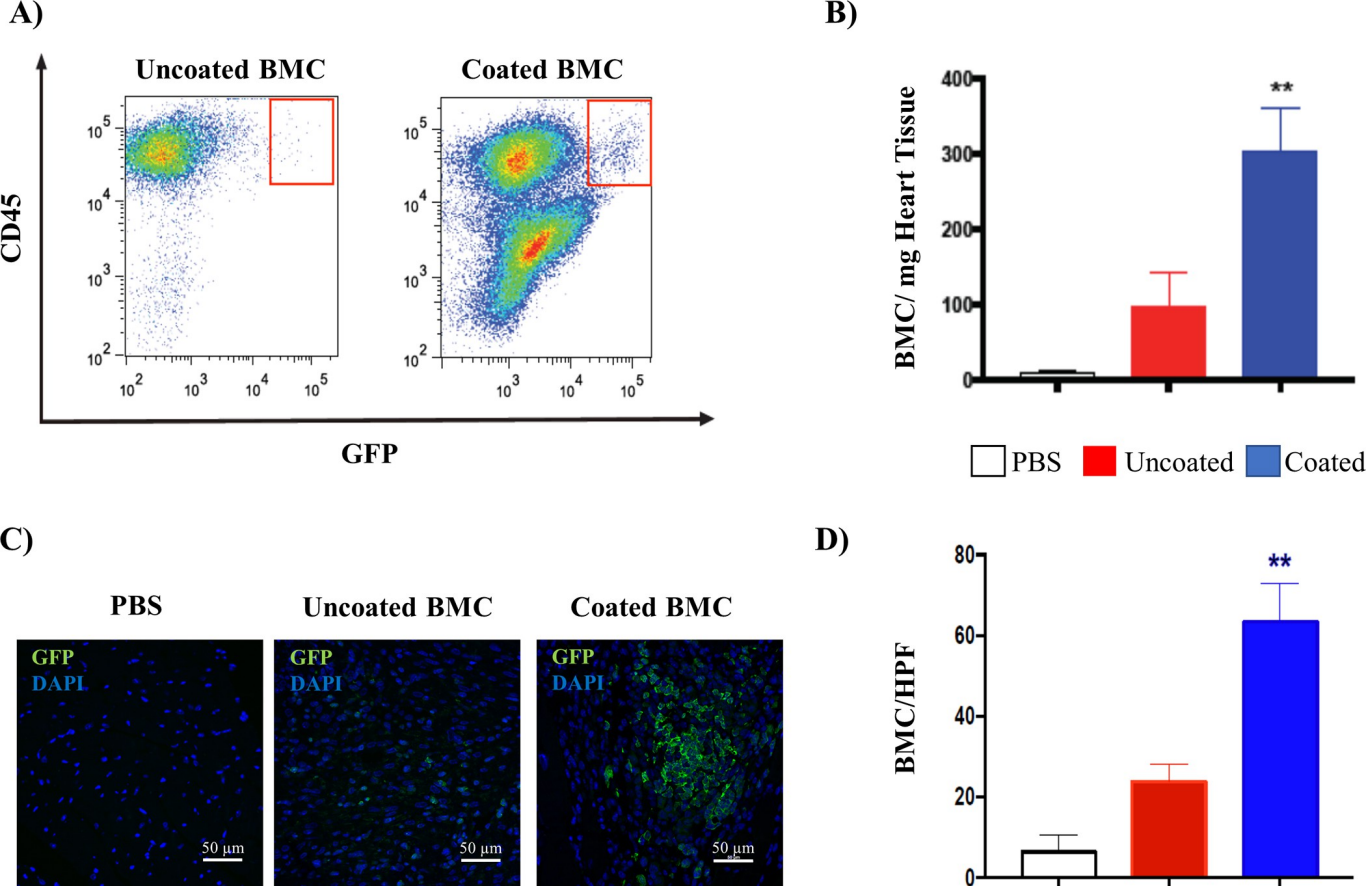
BMC retention. A) Flow cytometric analysis of digested heart tissue demonstrates a higher percentage of GFP^+^ cells in mice treated with coated cells compared to mice transplanted with uncoated cells. The majority of GFP cells were CD45^+^. B) Quantitative analysis of GFP^+^ BMC in digested heart tissue for coated versus uncoated cells by flow cytometry (signal in the PBS arm represents background). C) Immunohistochemical analysis demonstrated higher numbers of retained GFP^+^ BMC in mice treated with coated cells. GFP expression is shown in green with DAPI nuclear staining in blue. D) Quantitative analysis of GFP^+^ BMC in digested heart tissue for coated versus uncoated cells based on immunohistochemical analysis (signal in the PBS arm represents background). (N = 3 mice/group; *P<0.05, **P<0.01) (scale bar = 50 μm).

GFP^+^ BMCs were injected into the periinfarct region of a murine heart in a GFP^-^ animal. Retention was evaluated at seven days post-injection and stained for CD45 and GFP through flow cytometric analysis of the digested heart tissue. There was a significant increase in the retention of coated cells over uncoated cells in the digestate (uncoated = 96.5±45.3, coated = 302±58.9 cells/mg heart tissue, *P* = 0.0017, Fig 3A and 3B). Immunofluorescence analysis of tissue sections of the peri-infarct zone on day seven (Fig 3C and 3D) also demonstrated a significant increase in coated cell retention over that of uncoated cells (uncoated = 23.8±4.3, coated = 63.4±9.5 cells/high power field, *P* < 0.001).

### BMCs and coating interaction with representative cardiac cells

**Fig 4:**
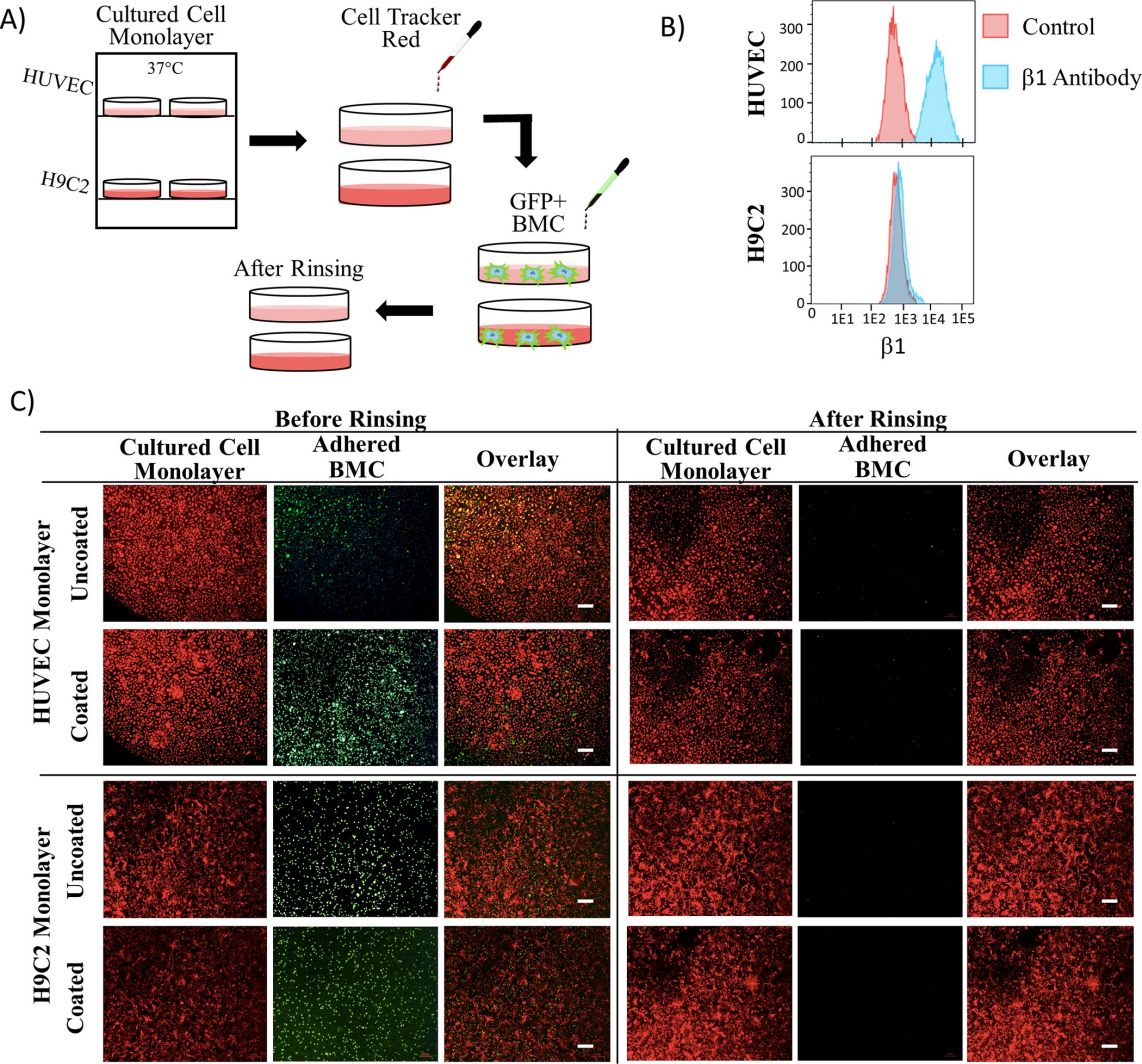
Expression of β1 integrins does not promote GelMA adhesion. A) Protocol for experiments examining the role of integrins in cell adhesion of GelMA coated cells. B) HUVEC and H9C2 cells analyzed by flow cytometry for β1 integrins by primary anti-β1 followed by secondary anti-AF647. C) Microscopic imagining of BMC incubated with cultured cells (HUVEC or H9C2) then subjected to rinsing by centrifugation at 800xg for 5 minutes. (n = 5 slides per test group). GFP expression of the BMC cells is shown in green and the HUVEC or H9C2 cells are shown in red using Cell Tracker Red.

Immunofluorescent staining of heart tissue showed β1 integrin labelling of resident cells (Fig 1B). Based on flow cytometric analysis (Fig 4B), HUVEC cells have high expression of β1 integrin, while the H9C2 cell line have low expression. Uncoated GFP^+^ BMCs were incubated with confluent monolayers of HUVEC or H9C2 cells, and few BMCs were retained after centrifugal rinsing (Fig 4C). Similarly, there was minimal retention of GelMA coated BMCs after incubation of confluent monolayers of HUVEC or H9C2 cells.

### BMC retention on extracellular matrix

**Fig 5:**
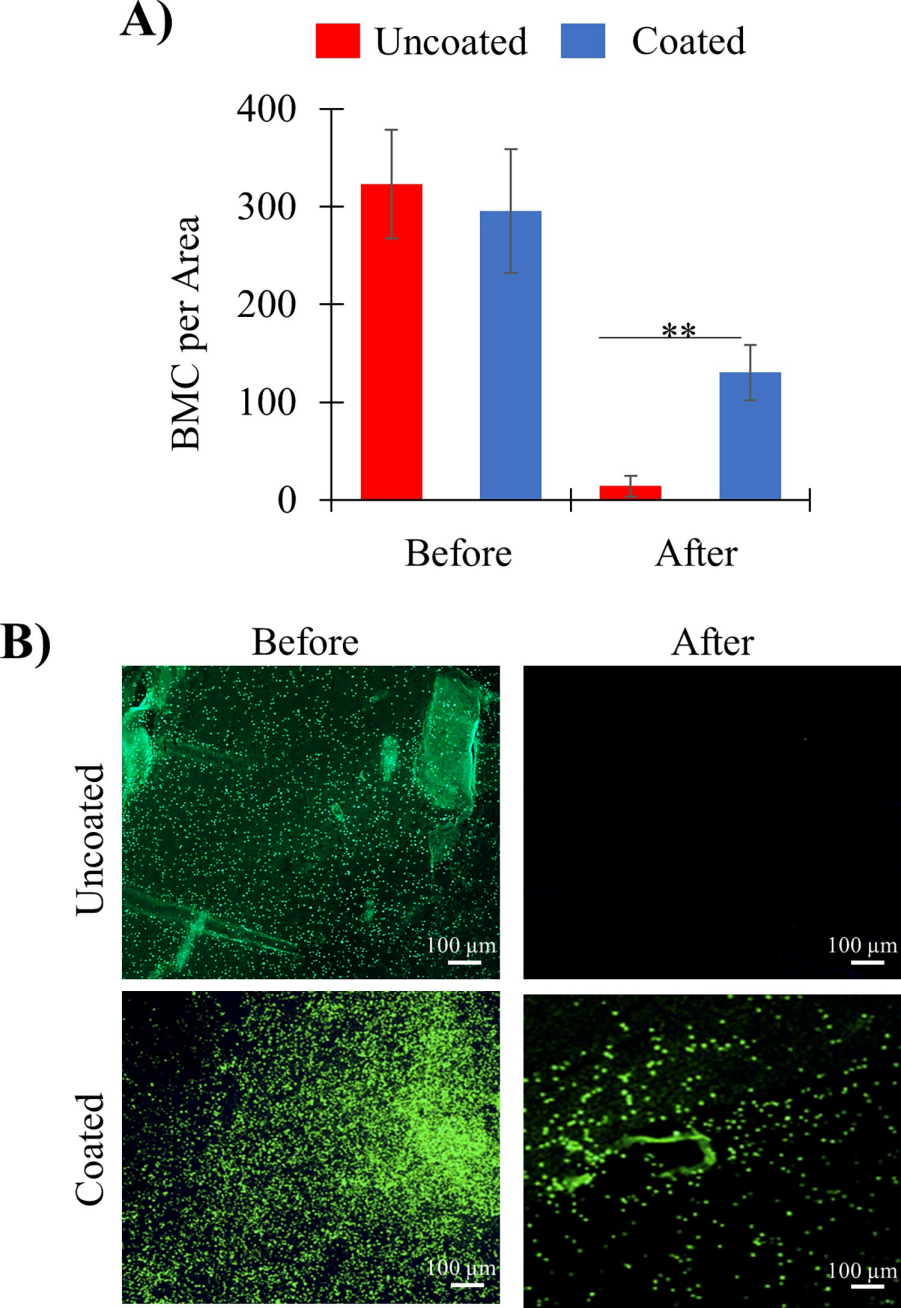
**A greater number of GelMA-coated BMCs are retained on decell heart tissue than uncoated BMC.** A) Quantitative analysis of the number of BMC per area determined by ImageJ before and after washing for coated versus uncoated BMC. B) Microscopic images of coated versus uncoated GFP^+^ BMC on decellularized tissue before and after rinsing. (n = 5 decell tissue per test group; p**<0.01) (scale bar = 100 μm).

After 40-minute incubation of GFP^+^ BMCs on decellularized heart tissue and centrifugal rinsing, a greater number of GelMA-coated cells were retained than uncoated cells (Fig 5A, p**<0.01). Coated BMCs were retained across the entire section of decellularized heart tissue (Fig 5B), while the uncoated cells were rarely observed. Retention of uncoated cells on decellularized tissue was exclusively limited to physical ridges in the decellularized tissue sections.

**Fig 6:**
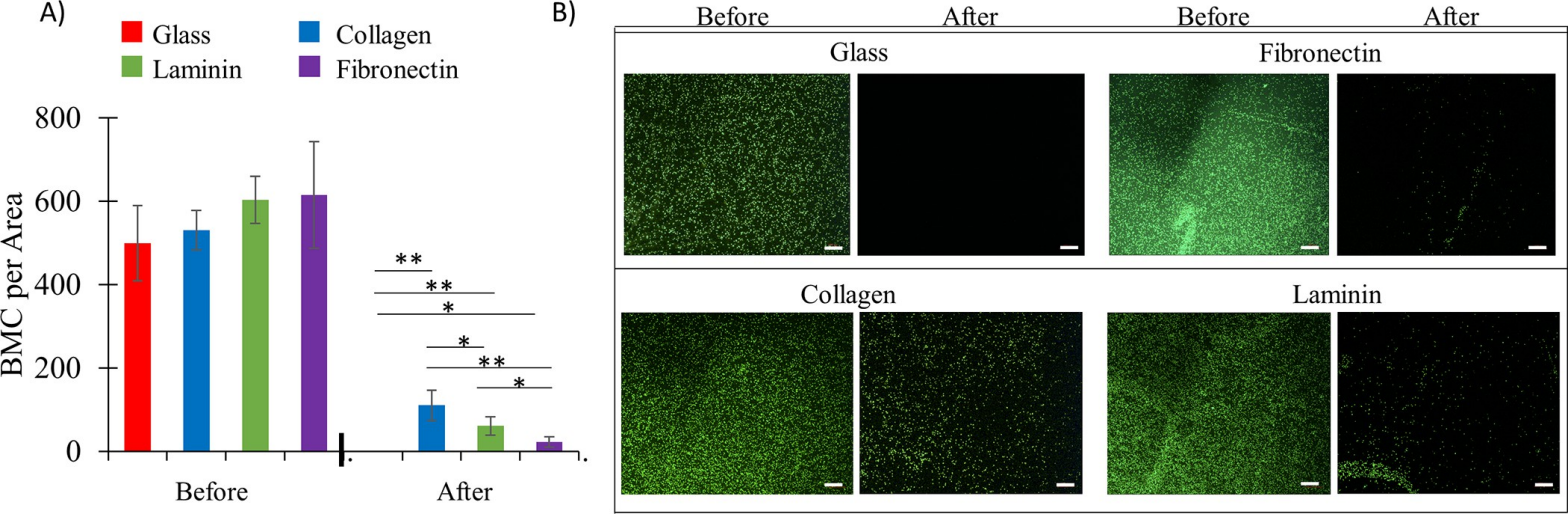
Retention of GelMA-coated BMC on glass substrates coated with purified ECM components. A) Mean number of BMC per area determined by ImageJ before and after washing for coated versus uncoated BMC. B) Microscopic images of GelMA coated GFP^+^ BMC on various ECM substrates before and after rinsing. (n = 5 slides per test group; p*<0.05, p**<0.01) (scale bar = 200 μm).

**Fig 7:**
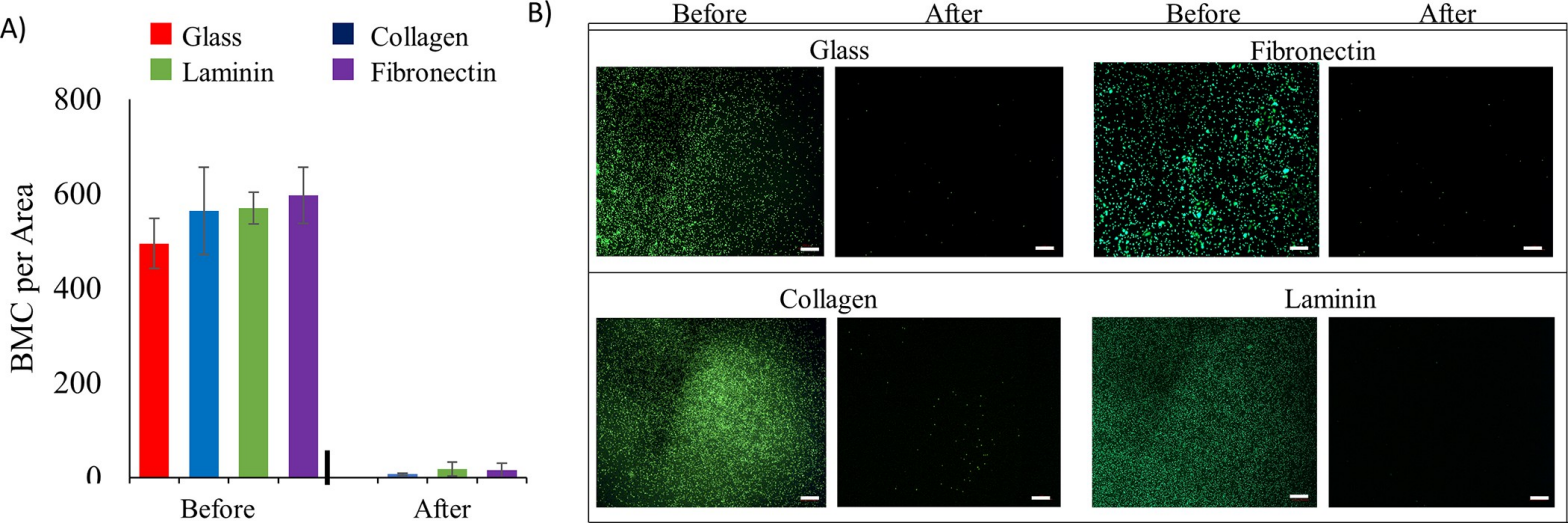
Retention of uncoated BMC on glass substrates coated with purified ECM components. A) Mean number of BMCs per area determined by ImageJ before and after washing for coated versus uncoated BMC. B) Microscopic images of uncoated GFP^+^ BMC on various ECM substrates before and after centrifugal rinsing. (n = 5 slides per test group; p*<0.05, p**<0.01) (scale bar = 200 μm).

GFP^+^ BMCs were contacted with purified ECM components of collagen, laminin and fibronectin. Unmodified glass was used as a control substrate in this study. Following a 40-minute incubation and centrifugal rinsing, all ECM substrates displayed increased retention of GelMA coated BMCs over that of a glass control substrate (Fig 6). Collagen-coated glass retained a higher density of the GelMA-coated BMCs than the laminin or fibronectin substrates (p < 0.05). When this ECM adhesion study was repeated using uncoated BMCs (Fig 7), there was minimal adhesion of the uncoated BMCs on collagen, laminin, or fibronectin surfaces.

## Discussion

Intramyocardial injection of therapeutic MSCs is intended to co-locate the therapeutic cells with the infarcted tissue for optimal modulation of the immune response and minimization of scar expansion. The local placement of therapeutic cells is foiled by the exceptionally poor retention of injected cells. We previously demonstrated that biodegradable GelMA coatings increased the retention of MSCs within the myocardium over uncoated MSCs [8]. Further, the injection of coated MSCs offered increased cardioprotection over the injection of uncoated MSCs [8, 15]. These patchy coatings of GelMA offer adhesion with the myocardial tissue while supporting intimate communication between the implanted cells and their environment. In Fig 3, these same GelMA coatings also increase the retention of unfractionated bone marrow cells, suggesting that the transplanted cell type is not a major factor in the enhanced retention of GelMA-coated cells. While these cellular coatings improve the retention of the cells and their resultant therapeutic effect, the mechanism of adhesion between the coated cell and the heart tissue has not been previously resolved. Our current study supports a better understanding of recent research and has taken an important step in delineating the function of GelMA *in vivo* and how it enhances the retention of transplanted cells in the cardiac environment.

The logical adhesion modes between coated cells are categorized into either coating-cell adhesion or coating-ECM adhesion. The methacrylated gelatin coatings are a functionalized derivative of collagen [16], and the abundance of natural cell-collagen interactions motivates the study of interactions between coated cells and integrins expressed on the surface of cells [17, 18]. The cells in the myocardium adhere to the ECM through integrins expressed on the surface of cells [17, 18], and β1 integrins are the major physiological receptors for collagen in cardiac tissue [19, 20]. As GelMA is derived from collagen [16], we explored the potential for gelatin-coated BMCs to adhere to β1 integrins within the infarcted myocardium. Cell monolayers of β1^+^ endothelial cells did not retain coated cells in our *in vitro* adhesion assays (Fig 4). To consider other nonspecific adhesion mechanisms, coated cells were incubated on monolayers of β1^-^ cardiomyoblasts, where again minimal adhesion was observed. While far from exhaustive, the minimal coated cell retention suggests the coating-cell interactions are not sufficient for adhesion of implanted cells to β1 integrin expressing cardiac cells or the resident endothelium. While previous studies have demonstrated the adhesion of cultured cells to GelMA scaffolds [21, 22], these models were not subject to fluid shear. Arterial wall shear stress is one of the highest physiological shear environments at 10–20 dyne/cm^2^ [23]. Our lab previously demonstrated MSC adherence to HUVEC through ICAM interaction. However, this adhesion could only withstand the arterial physiological shear when HUVEC were stimulated with TNF-α which increases ICAM surface expression [24]. This further supports our hypothesis that any short-term integrin-GelMA adhesion is too weak to withstand fluid shear.

Using this same *in vitro* adhesion assay, GelMA-coated cells showed a significant retention on decellularized ECM (Fig 5). These tissues have been stripped of resident cells, suggesting the remaining ECM components are a major driver for GelMA-coated cell retention. The lack of retention of uncoated cells on decell ECM (Fig 5) drives a distinction between the adhesion behavior of coated and uncoated cells on ECM. Taken together, the observations in Figs 6 and 7 are consistent with coating-ECM interactions driving the differential retention of coated cells and the uncoated cells during *in vivo* studies [15].

The GelMA-coated cells were most frequently retained when incubated on a collagen substrate. This study used type I collagen, and type I collagen accounts for over 80% of the collagen in the cardiac ECM [25]. Collagen subunits are well known to self-assemble into triple helices of procollagen, where they are later enzymatically crosslinked through lysine residues to form functional collagen fibrils [11]. As GelMA is formed from the hydrolytic degradation of collagen, it is reasonable to postulate the preservation of many structural elements that would promote the formation of procollagen. These noncovalent interactions may ultimately control the observed enhanced retention of gelatin-coated cells on collagen surfaces. This further substantiates our conclusion that BMCs alone do not promote adhesion within the myocardium, as evidenced by the litany clinical studies with poor retention of therapeutic stem cells [6, 26].

Myocardial infarction initiates a sterile inflammatory response that activates both the innate and humoral immune systems. While this response is important for clearing dead cells and preparing the heart to heal, it is an evolutionarily-blunt response and has been shown to exacerbate adverse cardiac remodeling and scar formation [3]. Throughout the first few weeks following MI, collagen expression in injured cardiac tissue increases two-fold [27]. This rapid increase in collagen expression is a potential therapeutic target for our GelMA-coated cells and explains their enhanced retention in our *in vivo* studies after MI.

## Conclusions

While transplantation of therapeutic stem cells has been greatly limited by poor uptake, gelatin-based cellular coatings allow for a significant increase in stem cell retention [8]. A recent meta-analysis of human clinical trials concluded that the number of therapeutic stem cells is positively correlated with therapeutic benefits post-MI [4]. GelMA is an appealing hydrogel for cell encapsulation due to its known biocompatibility, biodegradation, and tunable physical characteristics [16]. Through an *in vivo* mouse model, we demonstrated a 3-fold increase in BMC retention for coated versus uncoated cells (Fig 2). While cell imaging and flow cytometry strategies allow us to identify retained cells [8], they do not indicate what interactions are promoting this increased retention. We initially divided the potential adhesion sites into cell-based and ECM-based groups. In this work, we determine the increased retention of GelMA-coated cells inside the myocardium was attributed to favorable interactions between the GelMA coating and the collagen within the ECM.

## Supporting information

S1 Fig Images supporting analysis in Fig 4 of coated BMC on H9C2 after rinsing. (TIF)

S2 Fig Images supporting analysis in Fig 4 of coated BMC on H9C2 before rinsing. (TIF)

S3 Fig Images supporting analysis in Fig 4 of coated BMC on HUVEC after rinsing. (TIF)

S4 Fig Images supporting analysis in Fig 4 of coated BMC on HUVEC before rinsing. (TIF)

S5 Fig Images supporting analysis in Fig 4 of uncoated BMC on H9C2 after rinsing. (TIF)

S6 Fig Images supporting analysis in Fig 4 of uncoated BMC on H9C2 before rinsing. (TIF)

S7 Fig Images supporting analysis in Fig 4 of uncoated BMC on HUVEC after rinsing. (TIF)

S8 Fig Images supporting analysis in Fig 4 of uncoated BMC on HUVEC before rinsing. (TIF)

S9 Fig Images supporting analysis in Fig 5 of coated BMC on decellularized mouse heart tissue. (TIF)

S10 Fig Images supporting analysis in Fig 5 of uncoated BMC on decellularized mouse heart tissue. (TIF)

S11 Fig Images supporting analysis in Fig 6 of coated BMC on collagen. (TIF)

S12 Fig Images supporting analysis in Fig 6 of coated BMC on fibronectin. (TIF)

S13 Fig Images supporting analysis in Fig 6 of coated BMC on glass. (TIF)

S14 Fig Images supporting analysis in Fig 6 of coated BMC on laminin. (TIF)

S15 Fig Images supporting analysis in Fig 7 of uncoated BMC on collagen. (TIF)

S16 Fig Images supporting analysis in Fig 7 of uncoated BMC on fibronectin. (TIF)

S17 Fig Images supporting analysis in Fig 7 of uncoated BMC on glass. (TIF)

S18 Fig Images supporting analysis in Fig 7 of uncoated BMC on laminin. (TIF)

S1 File Data supporting analysis in Fig 3 of cell retention by flow cytometry and microscopy. (PDF)
