# Supplementary figures and images for: Gelatin coating enhances therapeutic cell adhesion to the infarcted myocardium via ECM binding
Source: PLoS One. 2022 Nov 10;17(11):e0277561. doi: 10.1371/journal.pone.0277561 (PMC9648752; doi:10.1371/journal.pone.0277561)

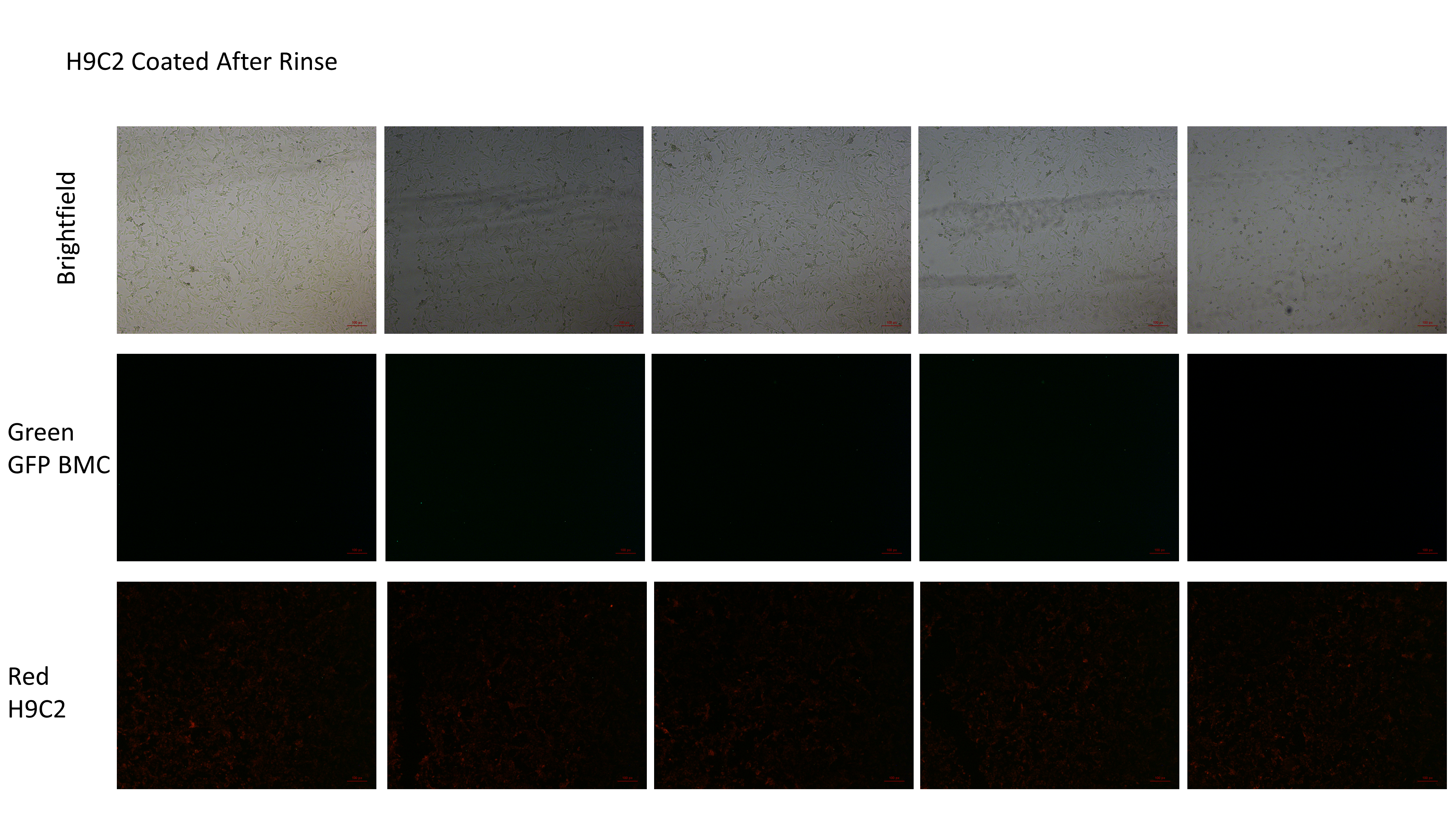

Supplement: S1 Fig — (TIF) [file pone.0277561.s001.tif]

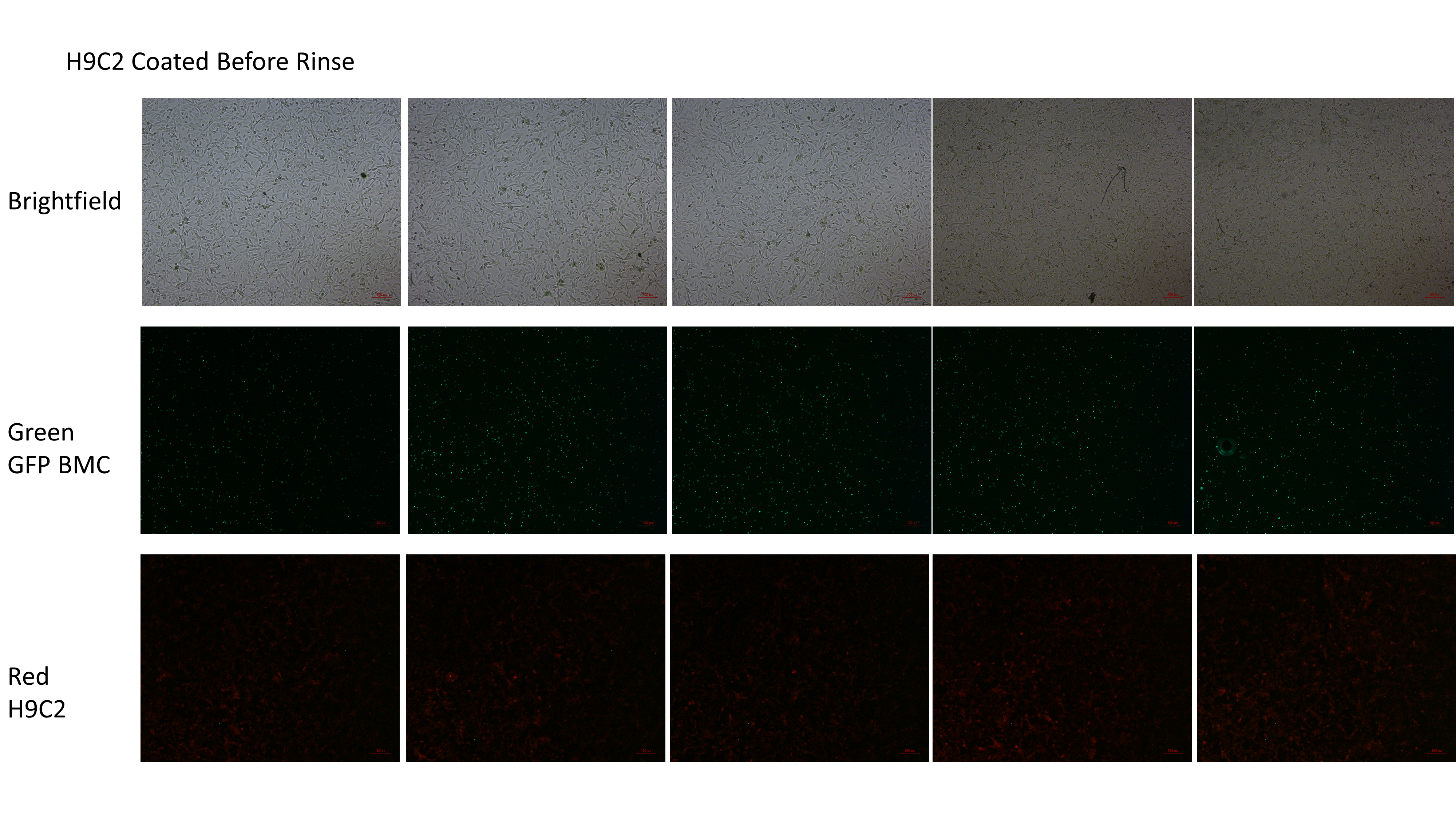

Supplement: S2 Fig — (TIF) [file pone.0277561.s002.tif]

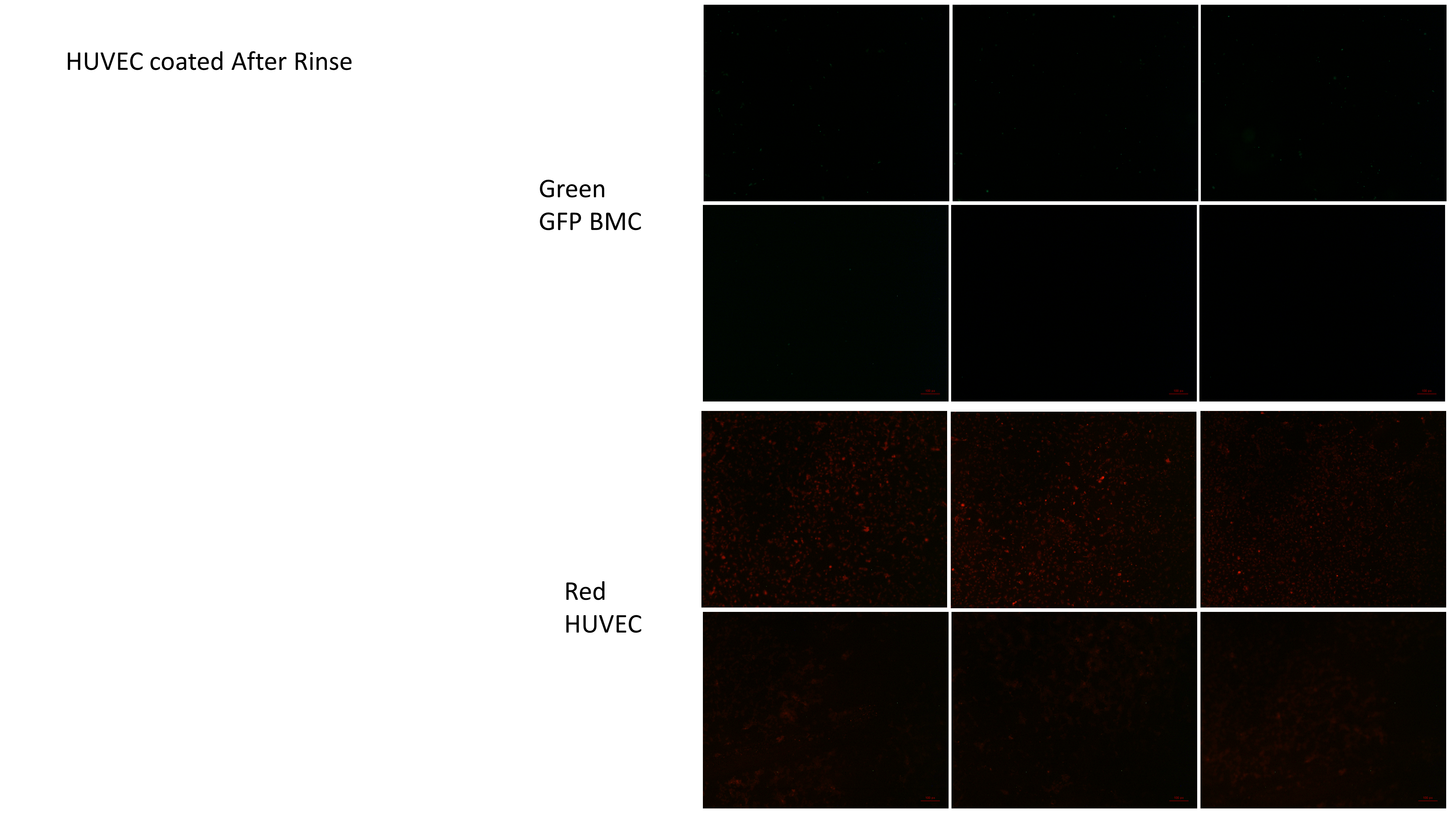

Supplement: S3 Fig — (TIF) [file pone.0277561.s003.tif]

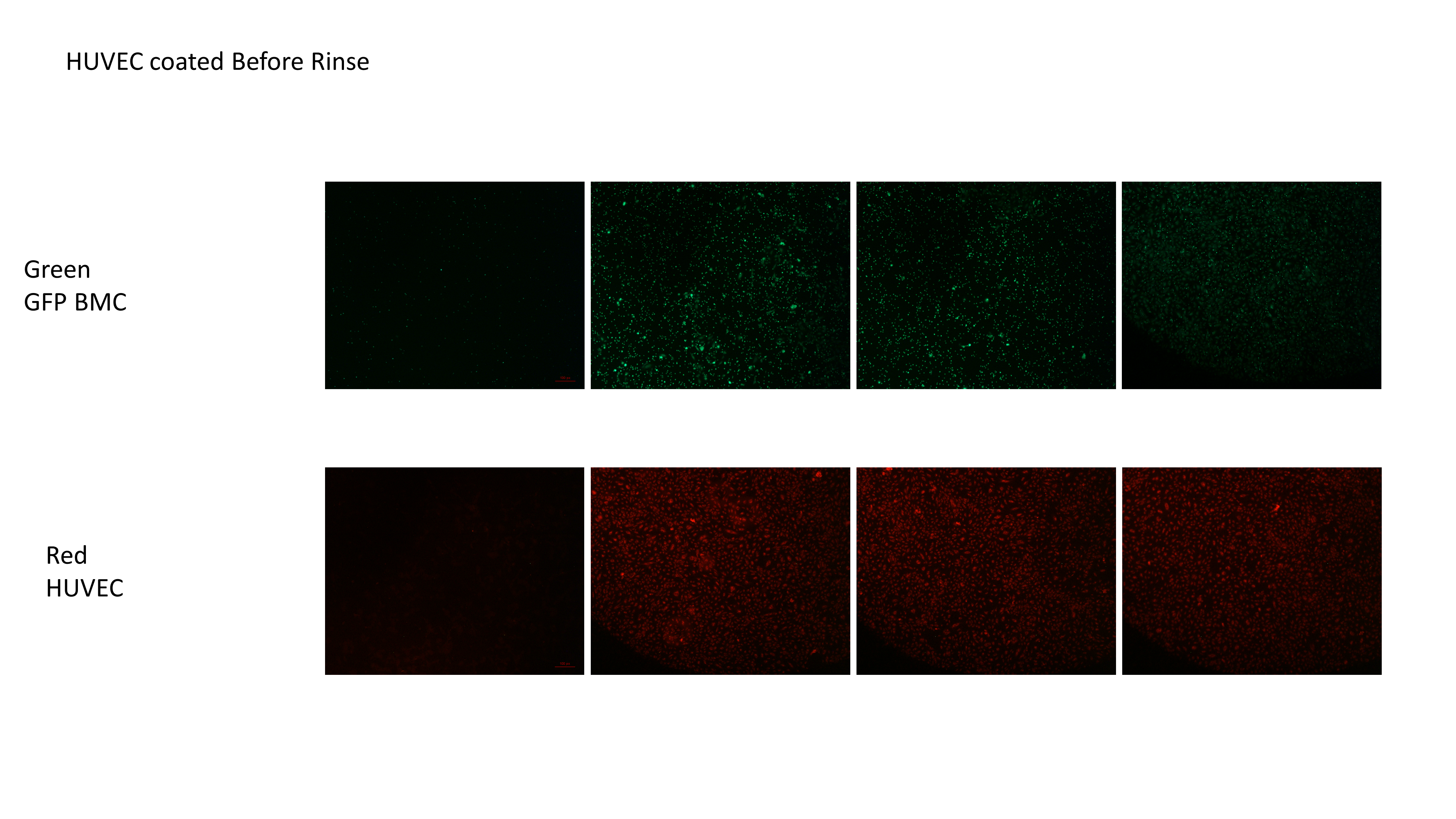

Supplement: S4 Fig — (TIF) [file pone.0277561.s004.tif]

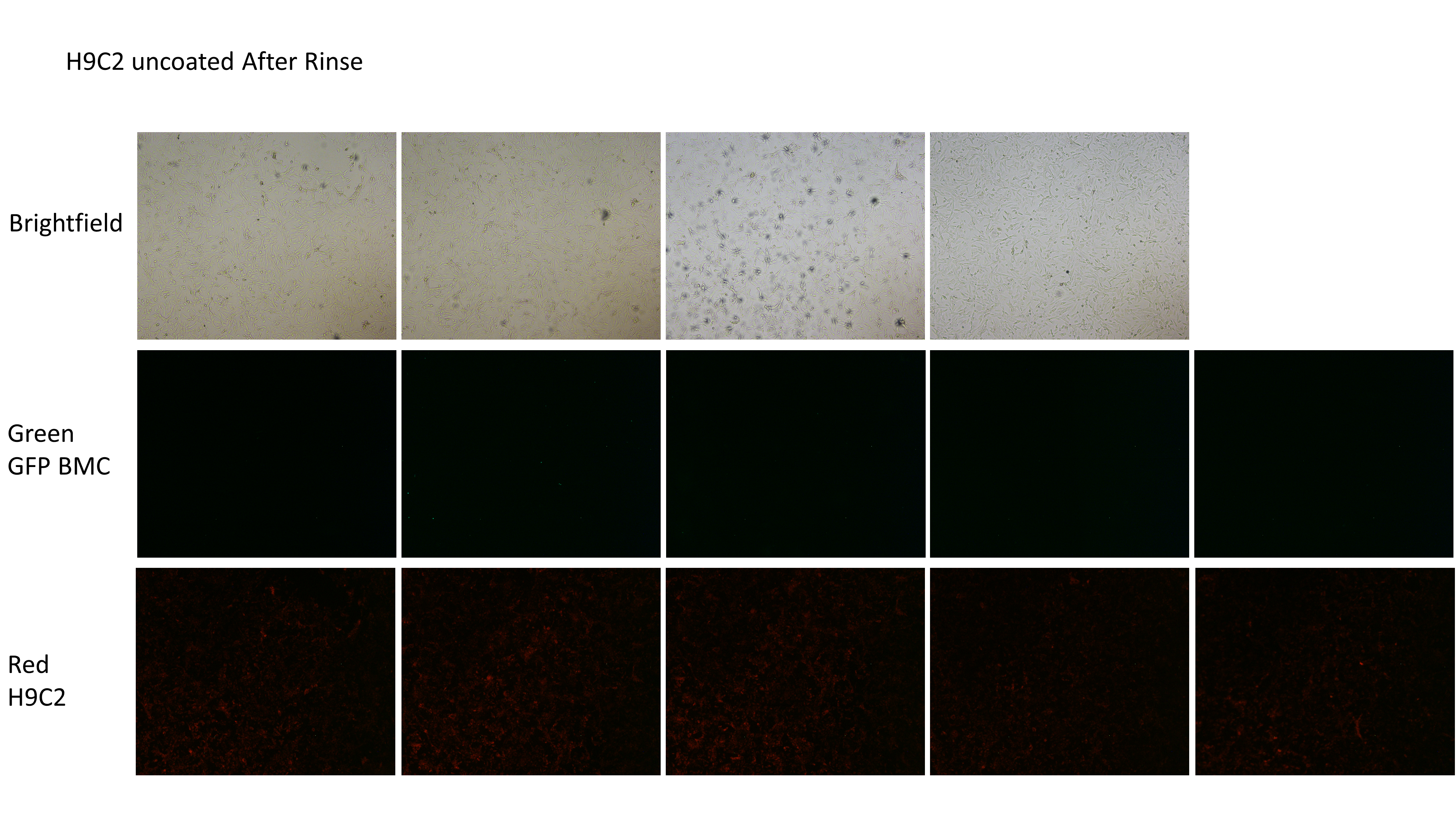

Supplement: S5 Fig — (TIF) [file pone.0277561.s005.tif]

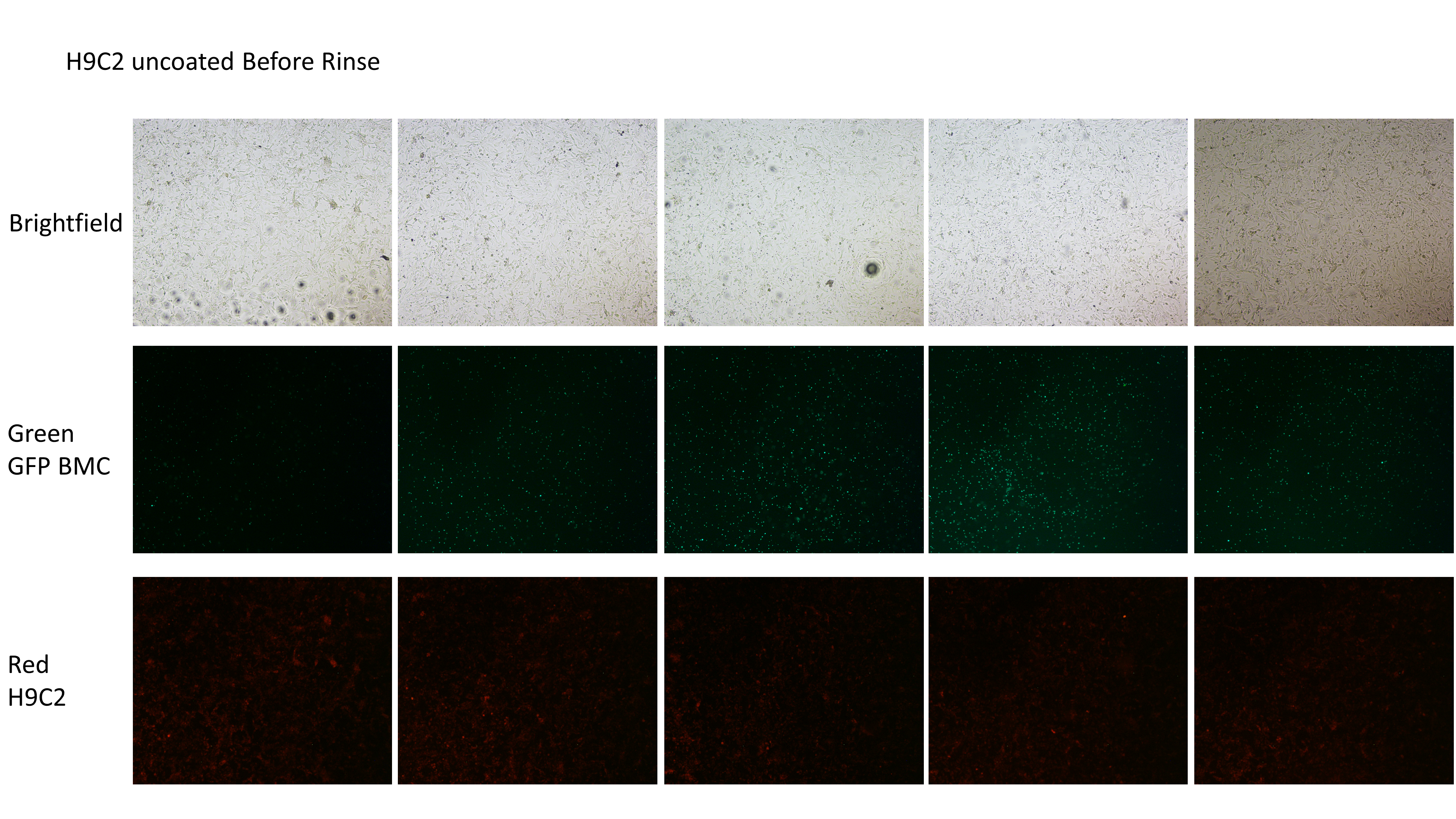

Supplement: S6 Fig — (TIF) [file pone.0277561.s006.tif]

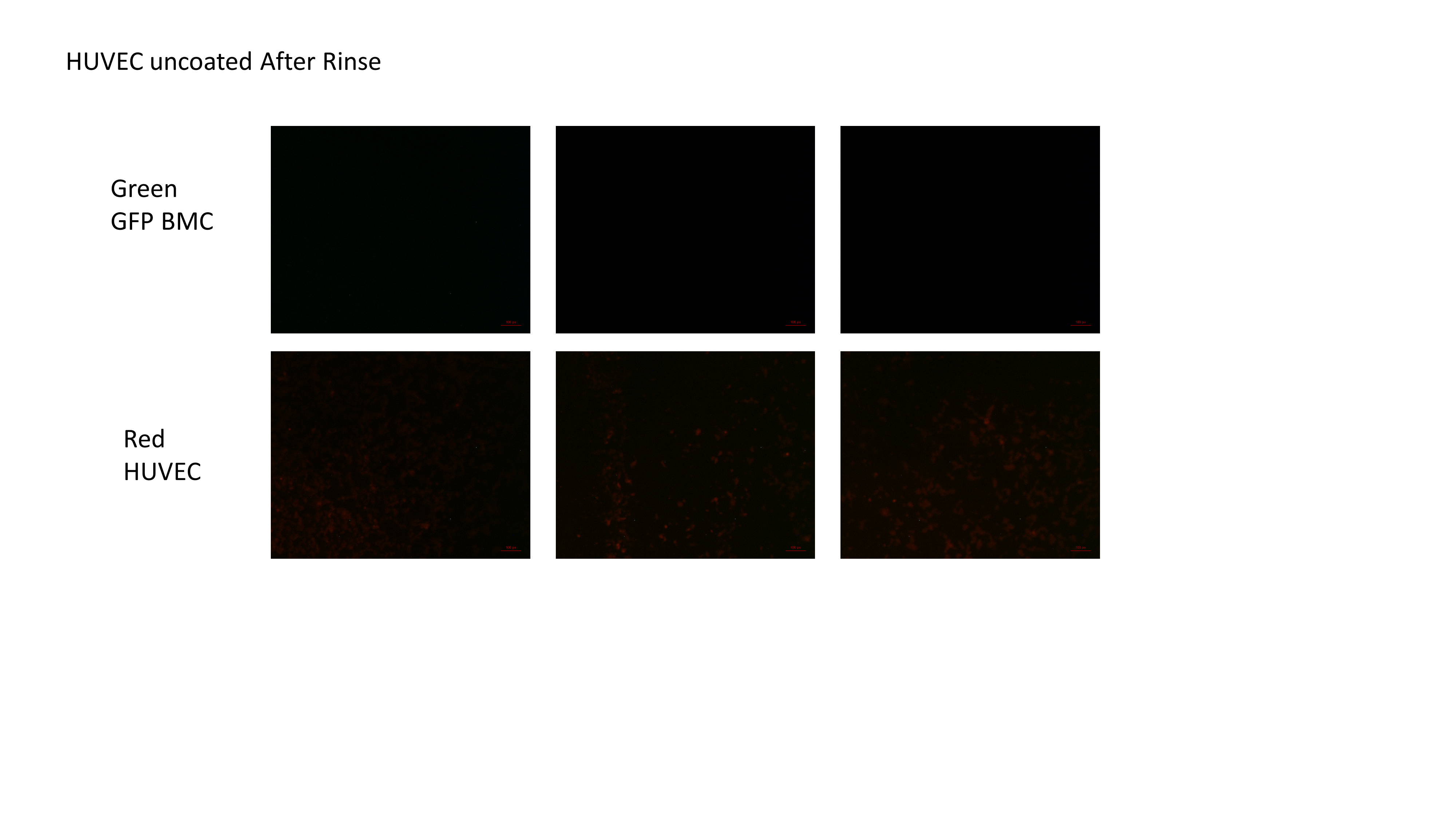

Supplement: S7 Fig — (TIF) [file pone.0277561.s007.tif]

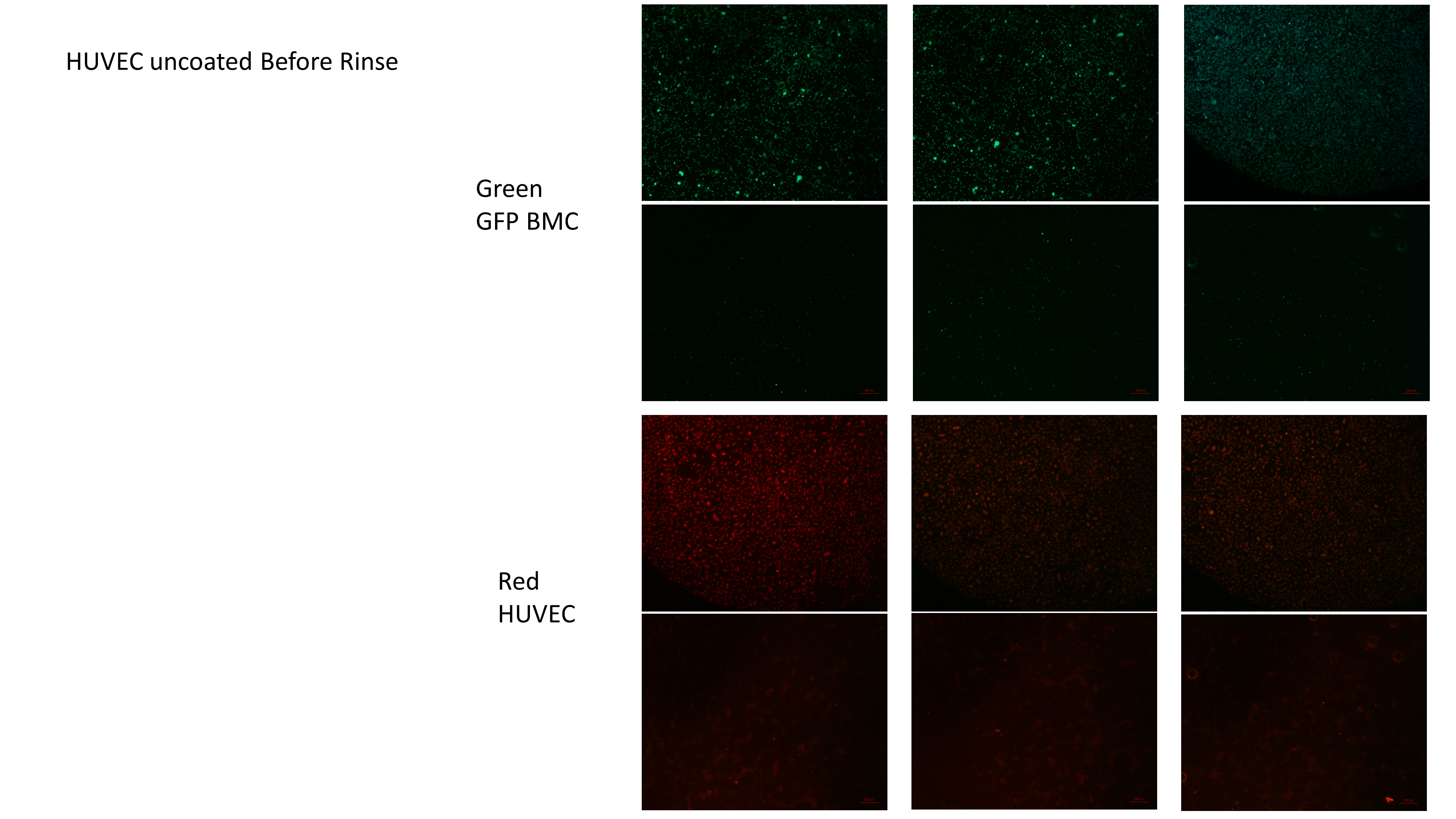

Supplement: S8 Fig — (TIF) [file pone.0277561.s008.tif]

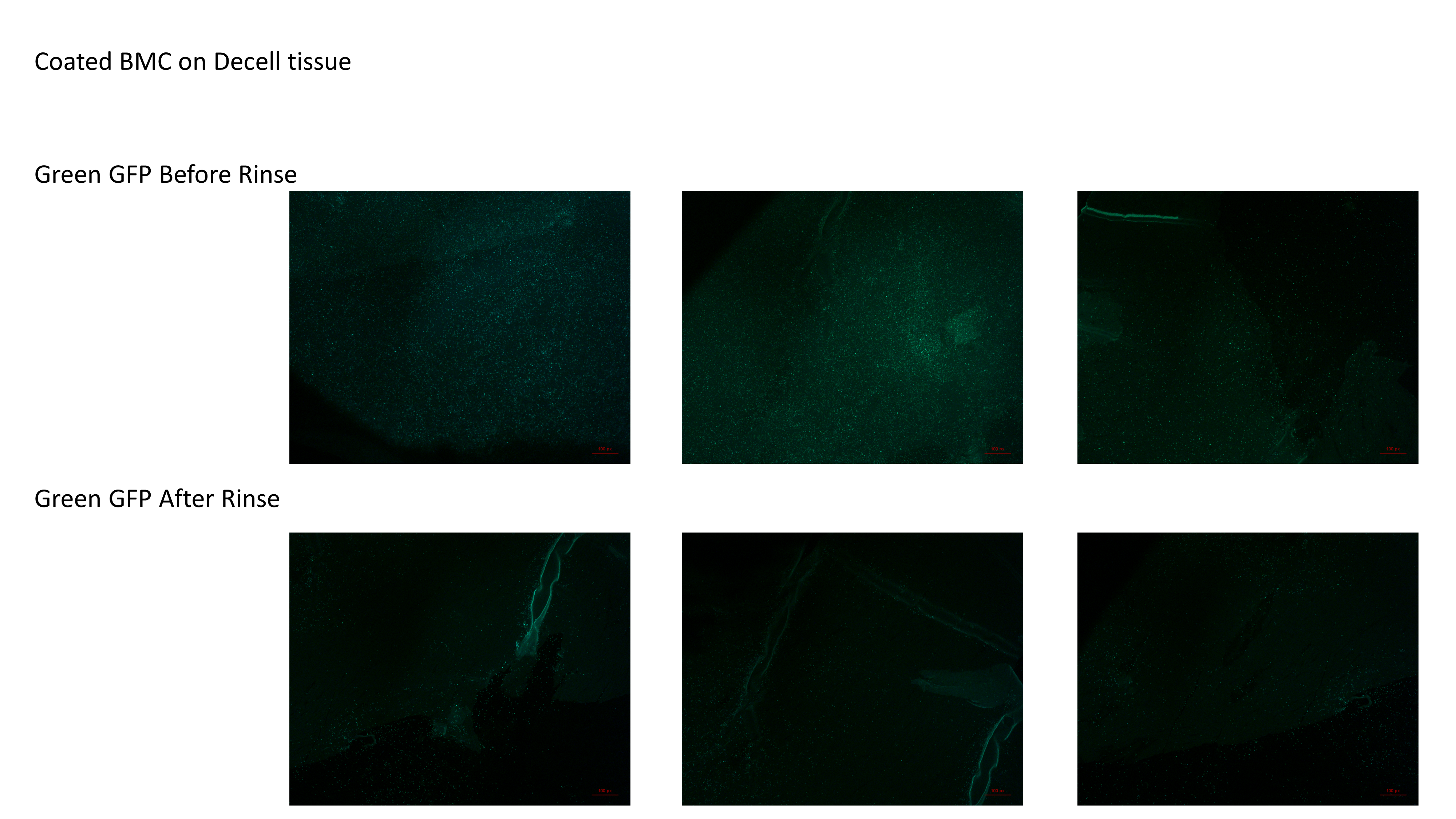

Supplement: S9 Fig — (TIF) [file pone.0277561.s009.tif]

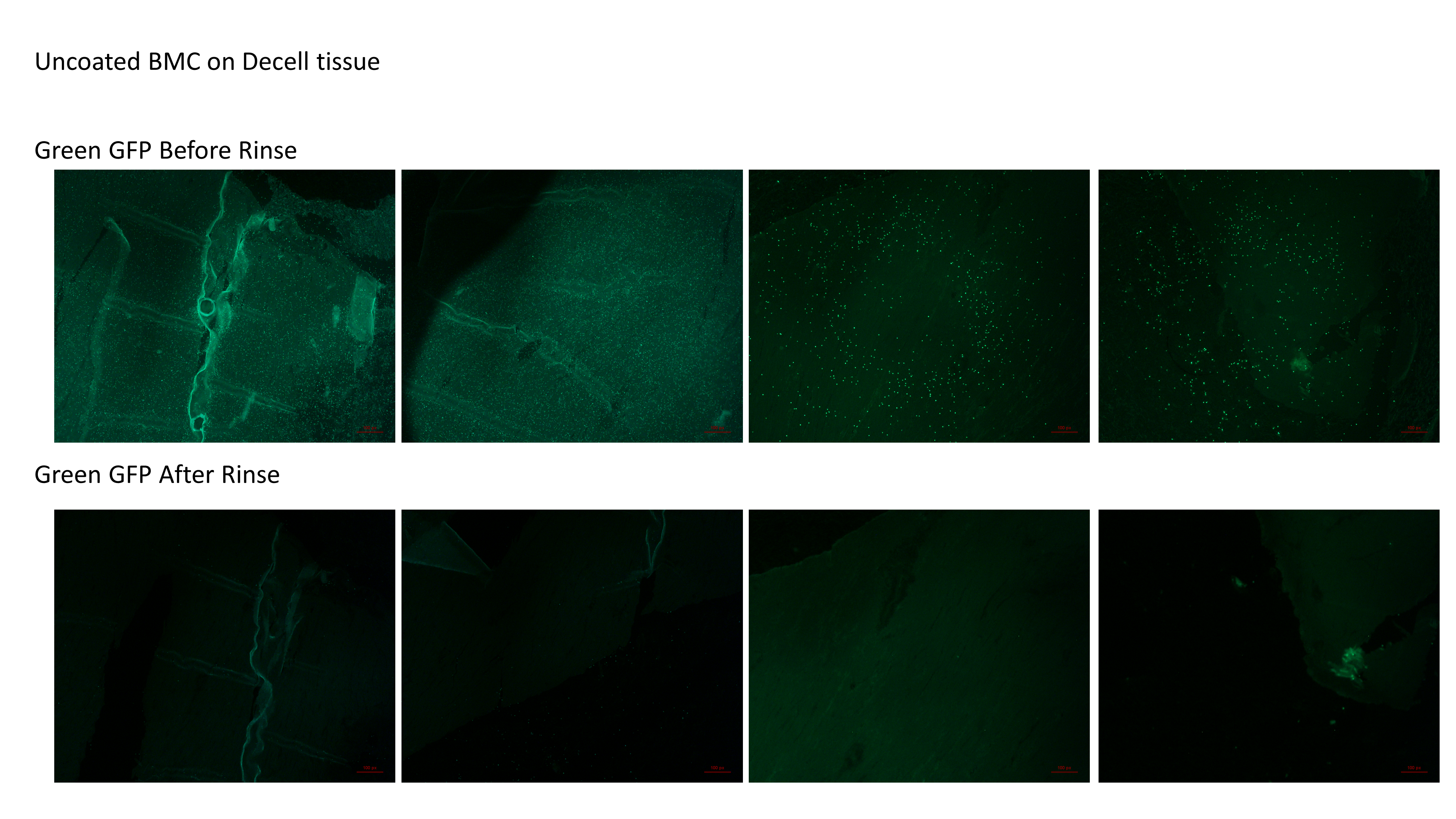

Supplement: S10 Fig — (TIF) [file pone.0277561.s010.tif]

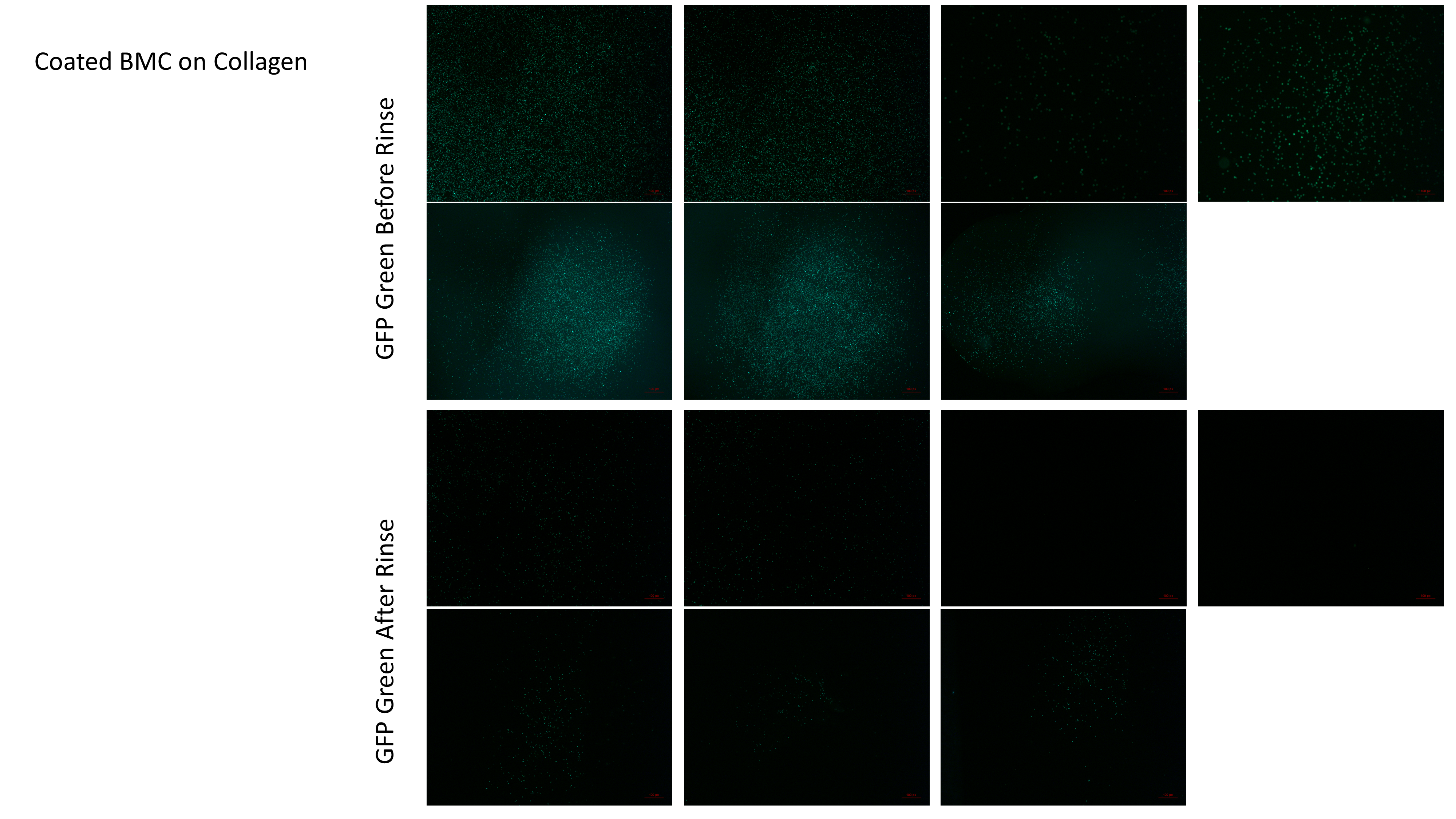

Supplement: S11 Fig — (TIF) [file pone.0277561.s011.tif]

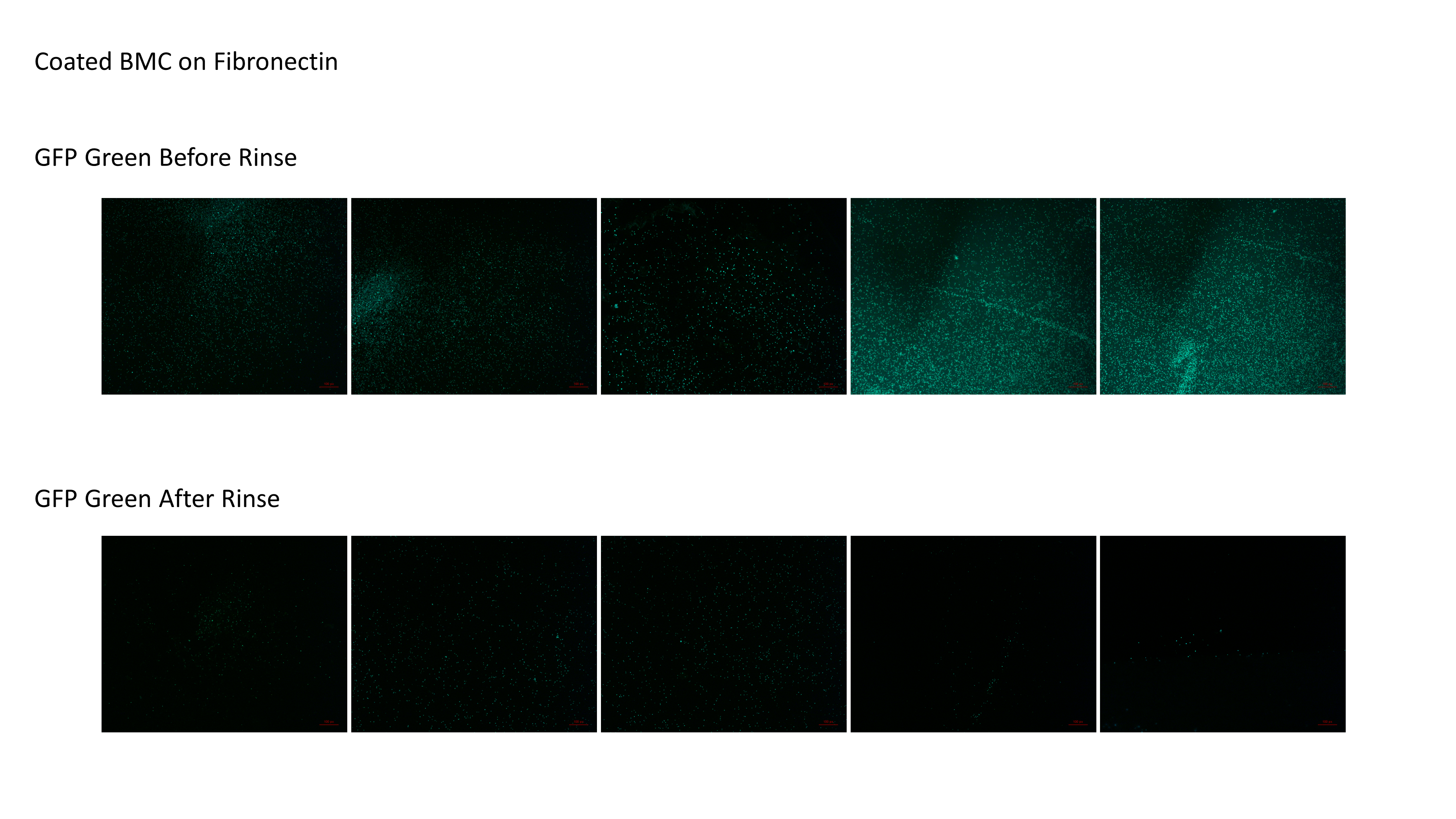

Supplement: S12 Fig — (TIF) [file pone.0277561.s012.tif]

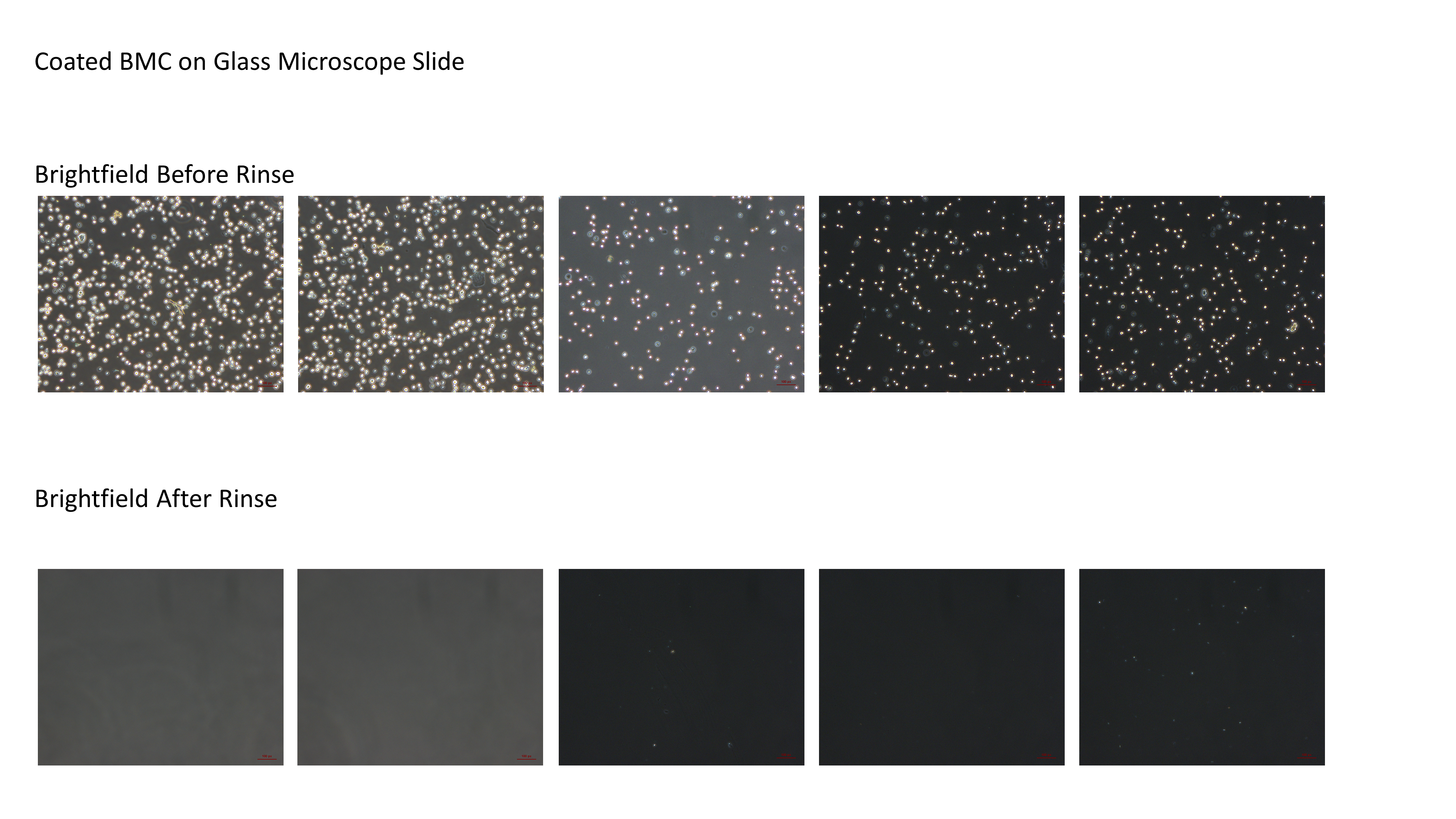

Supplement: S13 Fig — (TIF) [file pone.0277561.s013.tif]

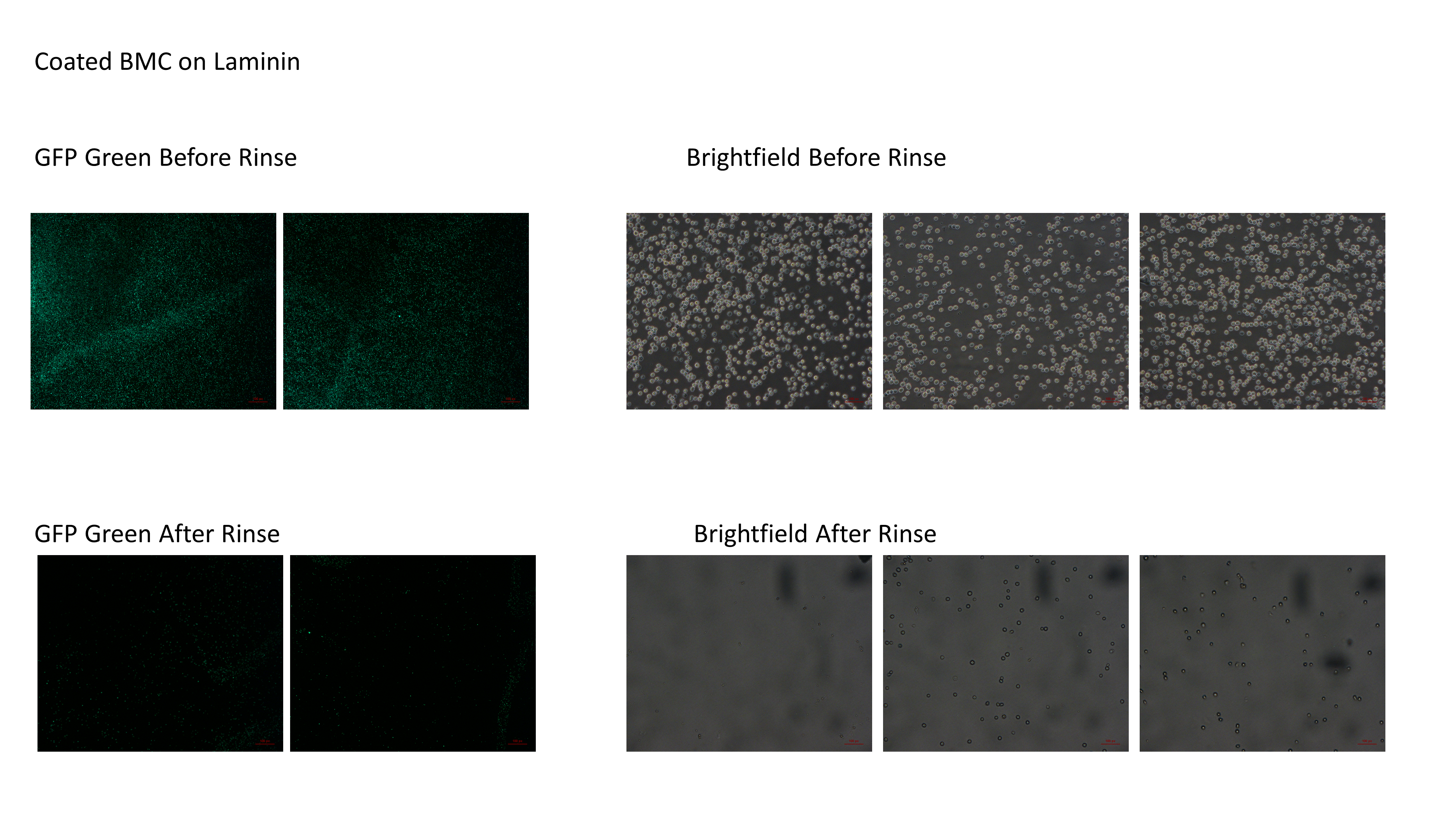

Supplement: S14 Fig — (TIF) [file pone.0277561.s014.tif]

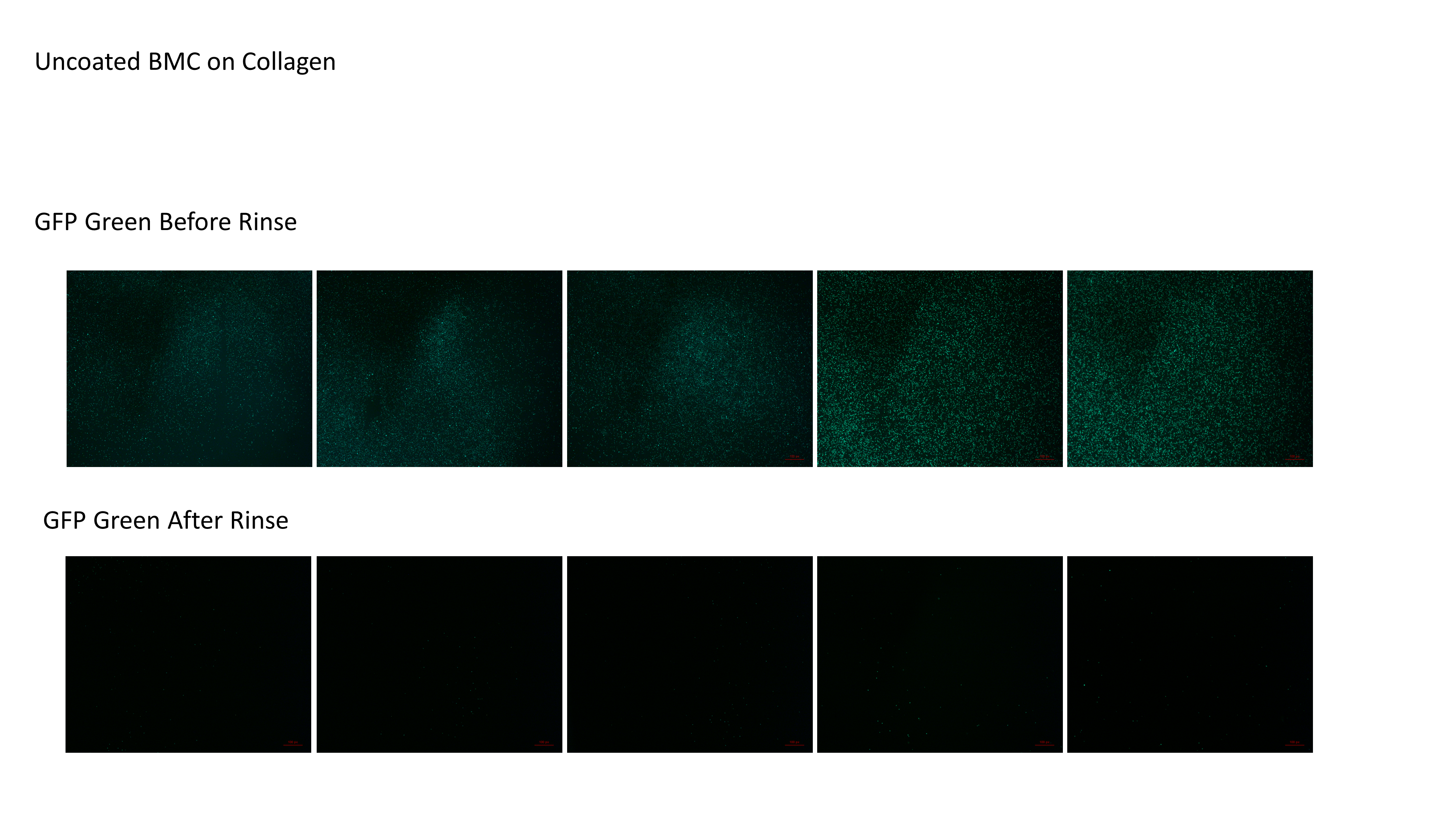

Supplement: S15 Fig — (TIF) [file pone.0277561.s015.tif]

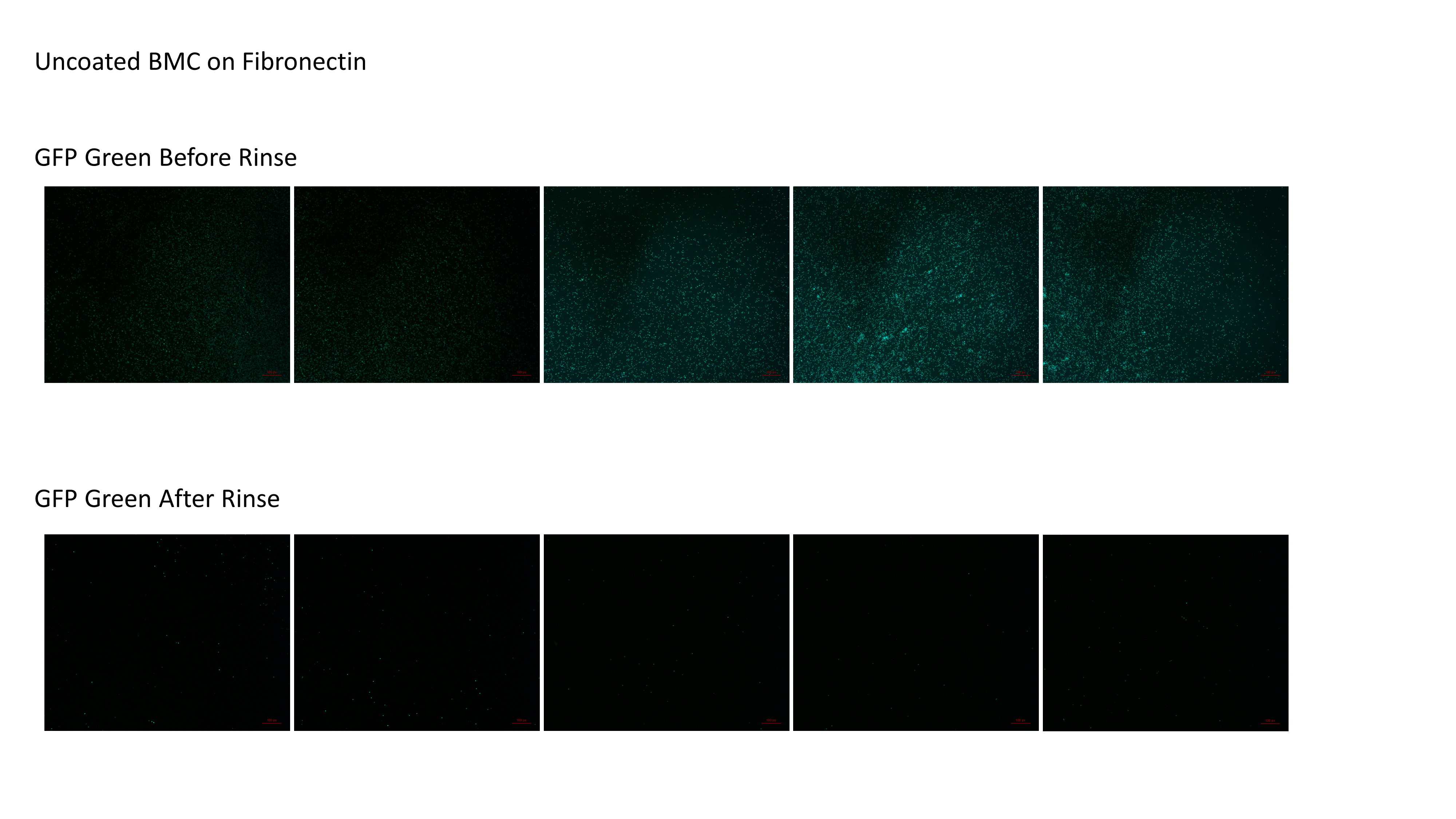

Supplement: S16 Fig — (TIF) [file pone.0277561.s016.tif]

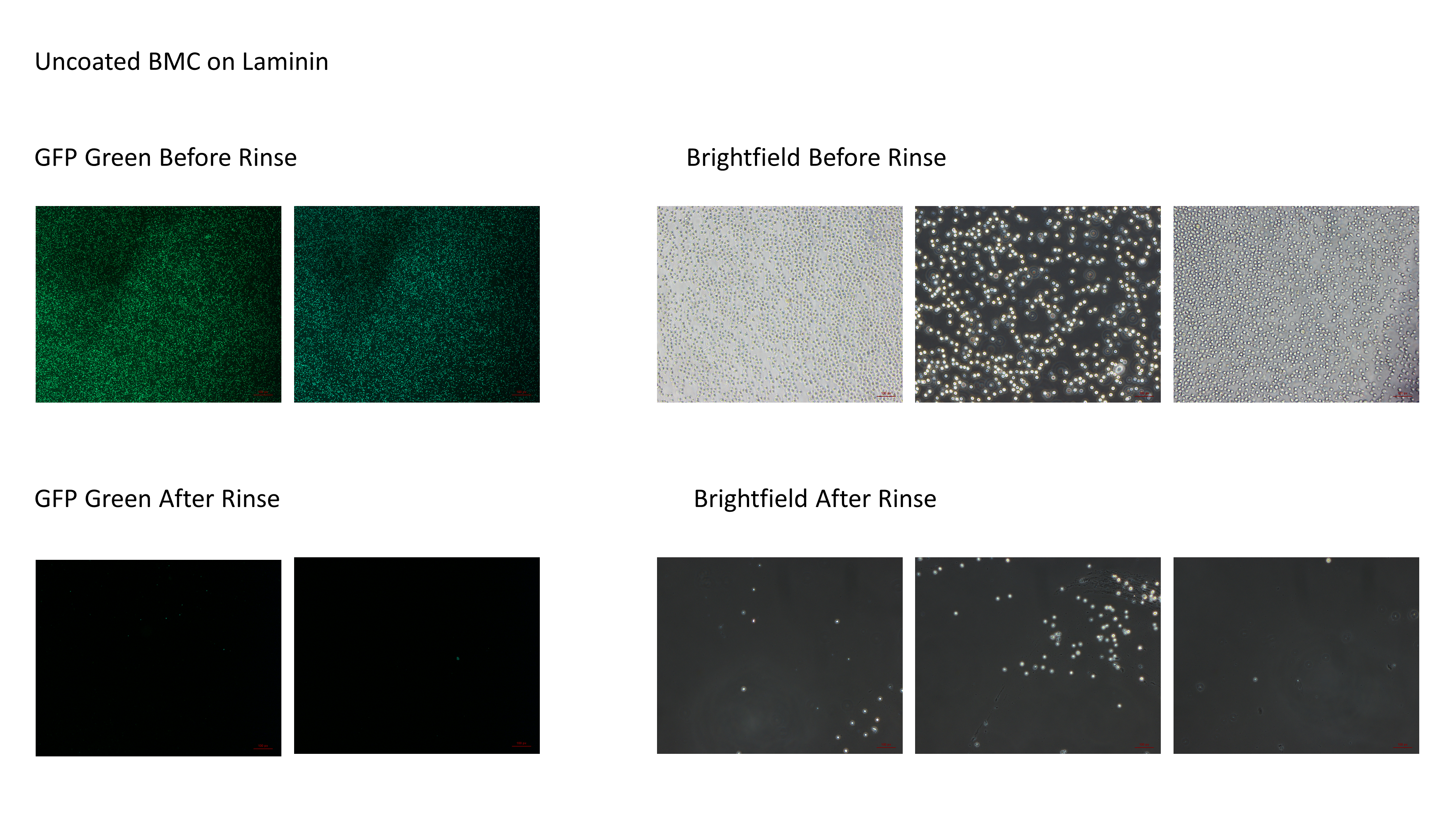

Supplement: S17 Fig — (TIF) [file pone.0277561.s017.tif]

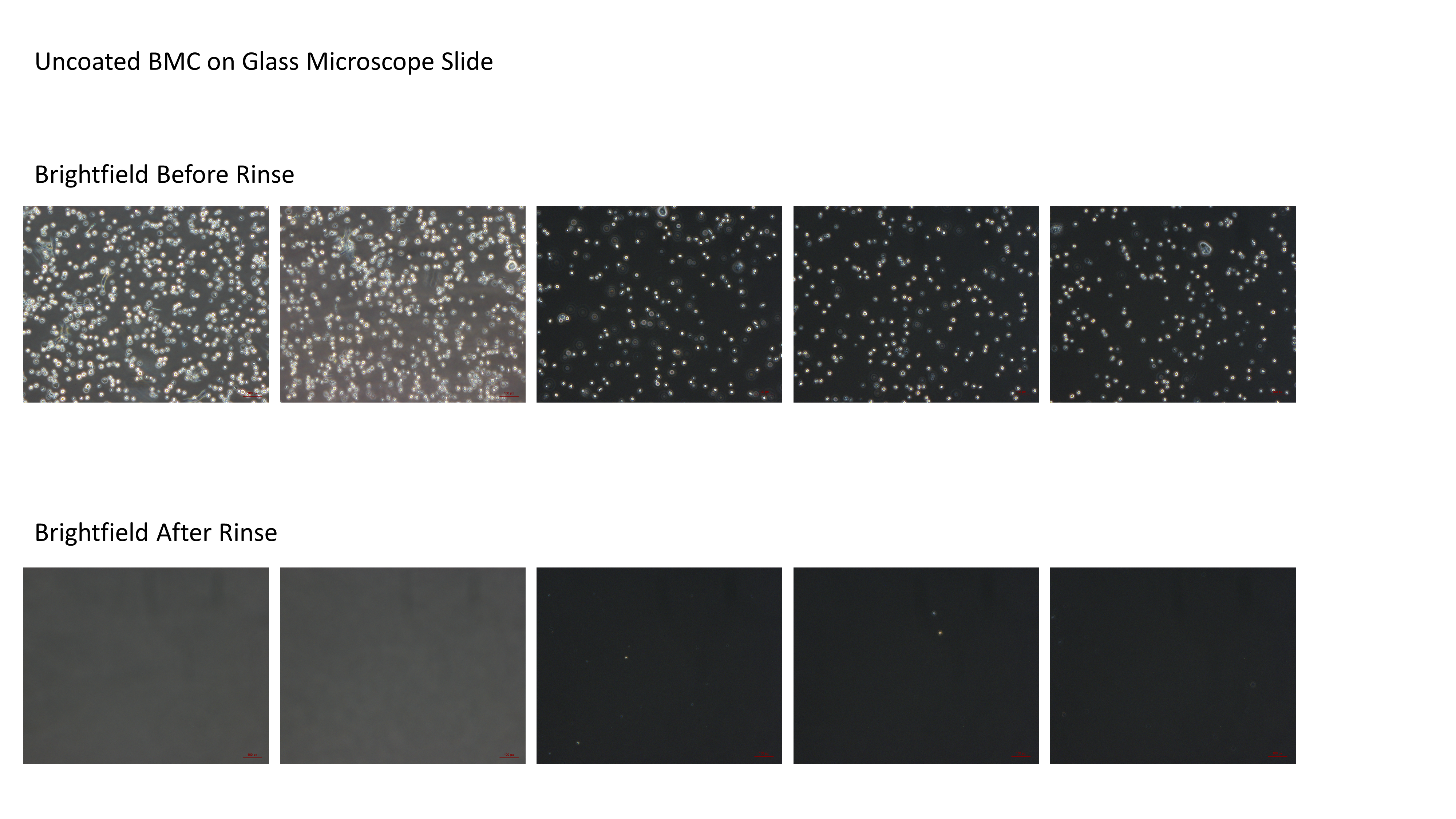

Supplement: S18 Fig — (TIF) [file pone.0277561.s018.tif]
