# Supplementary material for: Gelatin coating enhances therapeutic cell adhesion to the infarcted myocardium via ECM binding
Source: PLoS One. 2022 Nov 10;17(11):e0277561. doi: 10.1371/journal.pone.0277561 (PMC9648752; doi:10.1371/journal.pone.0277561)
Supplement: S1 File — (PDF) [file pone.0277561.s019.pdf]

Cell retention by histology

PBS	Uncoated Cells	Coated Cells
2	14	65
6	66	101
4	98	65
8	54	35
5	39	31
3	19	
0	7	95
	9	282
0	21	76
0	16	86
0	4	69
0	11	
2	16	15
0	14	26
60	21	44
	3	50
	13	21
		39
	15	53
	11	158
	17	33
	15	41
	79	48
	15	61
	23	39
	9	43
	32	29
	7	37
	17	55
	25	53
		88

Cell retention by flow cytometry

PBS	Uncoated Cells	Coated Cells
2	18	200
14	170	394
13	175	404
1	23	210
